# Organizing a global list of cyanobacteria and algae from soil biocrusts evidenced great geographic and taxonomic gaps

**DOI:** 10.1093/femsec/fiae086

**Published:** 2024-05-30

**Authors:** Luana Soares Dal-Ferro, Arthur Schenider, Dabny Goulart Missiaggia, Libério Junio Silva, Adaíses Simone Maciel-Silva, Cleber Cunha Figueredo

**Affiliations:** Departamento de Botânica, Universidade Federal de Minas Gerais, P.O. Box 486, 31270-901 Belo Horizonte, Minas Gerais, Brazil; Departamento de Botânica, Universidade Federal de Minas Gerais, P.O. Box 486, 31270-901 Belo Horizonte, Minas Gerais, Brazil; Departamento de Botânica, Universidade Federal de Minas Gerais, P.O. Box 486, 31270-901 Belo Horizonte, Minas Gerais, Brazil; Instituto Nacional de Pesquisas Espaciais, Divisão de Observação da Terra e Geoinformática (DIOTG), 12227-010 São José dos Campos, São Paulo, Brazil; Departamento de Botânica, Universidade Federal de Minas Gerais, P.O. Box 486, 31270-901 Belo Horizonte, Minas Gerais, Brazil; Departamento de Botânica, Universidade Federal de Minas Gerais, P.O. Box 486, 31270-901 Belo Horizonte, Minas Gerais, Brazil

**Keywords:** algae and cyanobacteria biogeography, biocrusts, ecological services, soil microorganisms, species global distribution

## Abstract

Biocrusts determine soil stability and resiliency, with a special role played by oxygenic photoautotrophic microorganisms in these communities. We evaluated temporal and geographic trends in studies focused on these microorganisms in biocrusts. Two databases were surveyed to obtain scientific articles published from 1998 to 2020 containing the terms ‘biocrusts,’ ‘algae,’ and ‘cyanobacteria.’ Although interest in biocrusts has increased recently, their ecological importance is still little explored. The scientific articles that mentioned a species list of cyanobacteria and/or algae revealed a very heterogeneous geographic distribution of research. Biocrusts have not been explored in many regions and knowledge in the tropics, where these communities showed high species richness, is limited. Geographic gaps were detected and more detailed studies are needed, mainly where biocrust communities are threatened by anthropogenic impacts. Aiming to address these knowledge gaps, we assembled a taxonomic list of all algae and cyanobacteria found in these articles, including information on their occurrence and ecology. This review is an updated global taxonomic survey of biocrusts, which importantly reveals their high species richness of oxygenic photoautotrophic microorganisms. We believe this database will be useful to future research by providing valuable taxonomic and biogeographic information regarding algae and cyanobacteria in biocrusts.

## Teaser

Biocrusts perform important ecological services, but they are little known. We generated a database revealing geographic and taxonomic gaps that will help further research on this topic.

## Introduction

Biological soil crusts (biocrusts) form a ‘living skin’ at the soil surface in many ecosystems around the world characterized by low primary productivity, including those severely limited by low water availability, nutrient scarcity, and cold temperatures (Belnap et al. 2001). Biocrusts are often the first community to colonize early-successional stages, resulting in changes that facilitate the establishment of other organisms throughout ecological succession (Zhang et al. 2016, Becerra-Absalón et al. 2019). This ability in determine communities establishment was evaluated in many studies focused on the ecological roles that biocrusts provide in natural environments, but recent researches are also evaluating how such communities can be managed for bioremediation of degraded soils (Antoninka et al. 2020).

Biocrusts can be composed of many different proportions of various oxygenic photoautotrophic groups (cyanobacteria, eukaryotic algae, lichens, mosses, or liverworts) (Belnap et al. 2016). They also support different assemblages of decomposers, as well as a faunal food web (Belnap et al. 2001). Terrestrial biocrusts occur in a variety of environments, from deserts to temperate and humid tropical climates, where they have important ecological roles because their component organisms are efficient carbon and nitrogen fixers in different environments/settings (Johnson et al. 2007, Strauss et al. 2012, Abrantes et al. 2023). By releasing organic compounds that are rich in carbon (C) and nitrogen (N), biocrusts act as a source of nutrients that support microbial populations, which are essential for the decomposition of soil organic matter (Sepehr et al. 2019). Furthermore, biocrusts are important for soil stabilization since they diminish wind and water erosion (Williams et al. 1995, Chamizo et al. 2017, Gao et al. 2020) and retain water, thereby maintaining higher humidity in soils (Delgado-Baquerizo et al. 2016, Baldauf et al. 2021). These effects contribute for the establishment and development of the soil biota, from microbes to plants (Antoninka et al. 2020).

Despite the importance of the ecological services provided by biocrusts, these communities are poorly studied, although interest has increased recently. Few studies of biocrusts were published prior to the 2000s, but a series of key events organized by the community of researchers dedicated to biocrusts has brought them from niche to mainstream. First, an initial publication in 2001 (Belnap et al. 2001) brought a great advance in biocrust research by demonstrating that they are a global phenomenon. A hastened internationalization of the topic followed, from a few research centers to the global scientific community that exists today. Another key event was the establishment, sustenance, and growth of a triennial international biocrust meeting, the International Workshop on Biological Soil Crusts, with four editions since 2010. Even with the recently increased focus on biocrusts, and the great advances mentioned above, there remains a lack of knowledge from a large-scale perspective (Bowker et al. 2018).

Biocrust species composition, spatial distribution patterns, and temporal trends (over time trends) are not widely studied nor well-known. Nonetheless, the limited literature on biocrusts reveals that the occurrence of each species depends on several factors, such as population size in a neighboring site, effectiveness of strategies for dispersion between sites and chances of finding favorable habitats when dispersing (Samolov et al. 2020). Part of the reason for the limited knowledge of oxygenic photoautotrophic organisms in biocrusts is that many studies have focused on cyanobacteria (currently phylum Cyanobacteriota) due the special roles these prokaryotes play in soil (Mazor et al. 1996, Roncero-Ramos et al. 2019, Abrantes et al. 2023). Since many cyanobacteria species can fix atmospheric N in biocrusts (Zhao et al. 2010), they are commonly considered to be very relevant in the first stages of ecological succession, contributing to the subsequent establishment of other organisms, such as some algae, lichens, fungi, mosses, and vascular plants (Weber et al. 2016, Szyja et al. 2019, Cantón et al. 2020). Nitrogen fixation and mechanisms to make this nutrient available to the surrounding environment (Abrantes et al. 2023) are relatively better known than the other ecological services biocrusts provide. Some aspects of N fixation have already been elucidated, such as its high dependence on temperature, water availability, light intensity, and species composition in biocrusts, since different species have different N-fixation rates (Zhao et al. 2010). As an ecological service, N fixation by biocrusts contributes to soil fertility in several regions, but mainly in the most N-limited arid and semiarid environments, since N is directly related to primary production and organic matter decomposition (Cantón et al. 2020).

Other contributions of biocrusts to environment functioning, beyond N fixation, have been described. Due to the common dominance of primary producers in biocrusts (mainly species of cyanobacteriota, algae, and bryophytes), C fixation occurs in relevant rates, which is especially important in biocrusts of arid regions where oxygenic photoautotrophic microorganisms also contribute to maintaining soil moisture (Steven et al. 2012, Cantón et al. 2020). In addition, cyanobacteria provide soil with resistance to erosion by water and wind, as they have exopolysaccharide sheaths that bind soil particles together, favoring the stabilization of the soil surface and the formation of soil aggregates (Roncero-Ramos et al. 2019, Cantón et al. 2020). This ecological role is specially observed in the genus *Microcoleus* (Belnap et al. 2001) and other non-nitrogen-fixing filamentous cyanobacteria, which commonly are among the first species that form new biocrusts in exposed surfaces. It is interesting to note that eukaryotic algae have potential to perform all or many of the functions mentioned above in relation to soil stabilization (Hashim et al. 2020), with the exception of N fixation. However, studies rarely focus on these microorganisms, and so their roles in biocrusts remain less understood than those of cyanobacteria.

Perhaps one of the most unknown aspects of biocrusts is their taxonomic composition, especially the fraction composed of oxygenic photoautotrophic microorganisms. It is surprising that there are so few scientific articles that describe or even only mention the species that are most commonly recorded in biocrusts from different ecosystems and their geographical distribution, since this information is essential for many scientific studies on biocrusts. In contrast to the vast literature for identifying aquatic cyanobacteria and algae, there are almost no complete taxonomic keys, books, or at least species lists for identifying these microorganisms in biocrusts. More information needs to be generated about the taxonomic composition of biocrusts since interest is increasing and its ecological roles, including effects on ecosystem resilience and potential for ecosystem recovery, could differ according to species composition (Miller et al. 2011, Samolov et al. 2020). Furthermore, some ecological services of biocrusts seem to be favored by species diversity. Hu et al. (2002), e.g. discovered that soil aggregation is more effective with biocrusts that contain many species than with those comprising just a single species. More studies focusing on biocrust services and the dependence of these services on biocrust species composition are necessary to evaluate the true role that these communities play in different ecosystems and biomes.

Thus, in this study we reviewed spatial and temporal patterns of studies that focused on species of free-living algae and cyanobacteria present in biocrusts. We also produced a complete list of all species of cyanobacteria and algae mentioned in these studies. The studies focused on symbionts in lichens or other organisms were not considered here since this could generate biased results because their ecological roles and taxonomy can be very different from those of microalgae and cyanobacteria. Furthermore, we used the geographical distribution of the records to elaborate a map and to reinforce the necessity to increase efforts to better understand the biocrusts of certain regions. The characteristics of the environments (local climate and type of substrate) where each of the most common species are found were summarized to try establish the typical habitat frequently occupied by taxa. The resulting database will be a valuable source of taxonomic information for future researchers.

## Methods

A data survey was carried out consisting of a detailed search for works published from 1998 to 2020 in which the topic biological crust was found together with the words algae and/or cyanobacteria. The searches were performed in Scopus, a scientific academic database, and in Google Scholar, a more popular and more accessible database. We evaluated temporal changes in the interest by this topic based on a more general survey, which was performed with the keywords ‘biological crust’/‘biocrust’ and ‘autotrophic microorganisms,’ ‘Biological crust’/‘Biocrust,’ and ‘algae’ or ‘Biological crust’/‘Biocrust’ and ‘cyanobacteria’ at 1-year intervals. Searches with Google Scholar detected all kinds of works, including Doctoral and Masters theses, texts for scientific dissemination and scientific articles, while those with Scopus were restricted to scientific articles. We have also performed a more complete survey, including searches for more specific keywords: ‘biological crust’/‘biocrust’ and ‘diatoms,’ ‘biological crust’/‘biocrust’ and ‘bacillariophyta,’ ‘biological crust’/‘biocrust’ and ‘chlorophyta’/‘chlorophyte,’ ‘biological crust’/‘biocrust’ and ‘chlorophyceae,’ ‘soil biofims’ and ‘diatoms,’ ‘soil biofims’ and ‘bacillariophyta,’ ‘soil biofims’ and ‘chlorophyta’/‘chlorophyte,’ and ‘soil biofims’ and ‘chlorophyceae.’ This more specific survey contributed to an increase of approximately 10% in the list of research on the topic, and the complete list (general plus the most specific surveys) was used in spatial analyses and to compare the interest in different taxonomical groups.

We focused only on scientific articles, since we believe there is greater standardization of methods as they are peer-reviewed texts. Scientific articles obtained from the two databases were used to synthesize information about the species composition of algae and cyanobacteria present in biocrusts. This taxonomic information was organized in a table containing the following information about each study: authors, year of publication, species identification method, species present, country where the study was performed, and title (Supplementary Material - S1). The table was prepared using only studies in which the species of algae and cyanobacteria were actually observed and identified in biocrusts, and excluded those lacking a taxonomic survey or focusing on species that were only used as experimental models. Two other tables (Supplementary Material - S2 and S3) were organized to present details on the ecological conditions mentioned in the studies, mainly related to the region, local climate, and substrates where the most common species were found.

Beyond general temporal trends, a more specific evaluation was performed from a spatial perspective, considering the geographical distribution of the studies that focused on species of algae and cyanobacteria that compose biocrusts. This information was synthesized in a gradient world map that was prepared with the program Qgis 3.16 using the number of papers citing species by country.

Taxonomic information was evaluated from four perspectives. The first was from a broader view, using the dataset to detect the most commonly studied group of oxygenic photoautotrophic microorganisms in biocrusts, based on the number of articles that focused only on algae, only on cyanobacteria or on both groups. The second perspective used all the articles containing a species or genera list to synthesize taxonomic information obtained at the level of genus or species. If a genus had species identified in some studies but not in others, the information was presented as independent taxon reports for both. Two tables were prepared containing a list of names of the most commonly reported (at least in three studies) species of cyanobacteria and algae along with the countries and regions (tropical, temperate, or polar) where each were collected. It is important to highlight that these three geographic regions were established exclusively based on latitude, without considering more refined climatic differences or social and political aspects. Further refinement of the local climate has been organized in other databases (Supplementary Material S2 and S3), which is discussed elsewhere in this text. Thus, tropical region was considered the area between the Tropic of Capricorn and the Tropic of Cancer; temperate, between the tropics, and Polar Circles; and polar region was considered the regions within Polar Circles). The third perspective evaluated here was the pattern of species richness for algae and cyanobacteria between tropical and temperate regions, to determine whether highly diverse communities were being neglected. Comparisons between tropical and temperate cyanobacteria and algae richness were performed by Kruskal–Walis test (Past 4.03 Software), due to data heteroscedasticity. We focused on latitudinal patterns, as we previously detected a large difference in the number of studies between temperate and tropical regions. Although this could be considered not adequate to describe the ecology of biocrusts, we use it since this latitudinal distribution of research seems to overcome the real greater importance of habitat characteristics in determining the composition, richness, and diversity of species. Thus, the characterization of habitats was also done here as the fourth perspective, but discussed with less emphasis. For the fourth perspective, we have prepared a synthesis about the substrate and local climate where each more common taxon was found to resume information on its major ecological characteristics.

Our database was built considering the taxa as cited by authors. However, we used two updated databases (CyanoDB 2.0, accessed in http://www.cyanodb.cz; and Algaebase, accessed in https://www.algaebase.org/) to check if the names are currently accepted taxonomically. Names have been updated and, in cases of small spelling errors in the articles, they have also been corrected.

## Results

**Figure 1.**
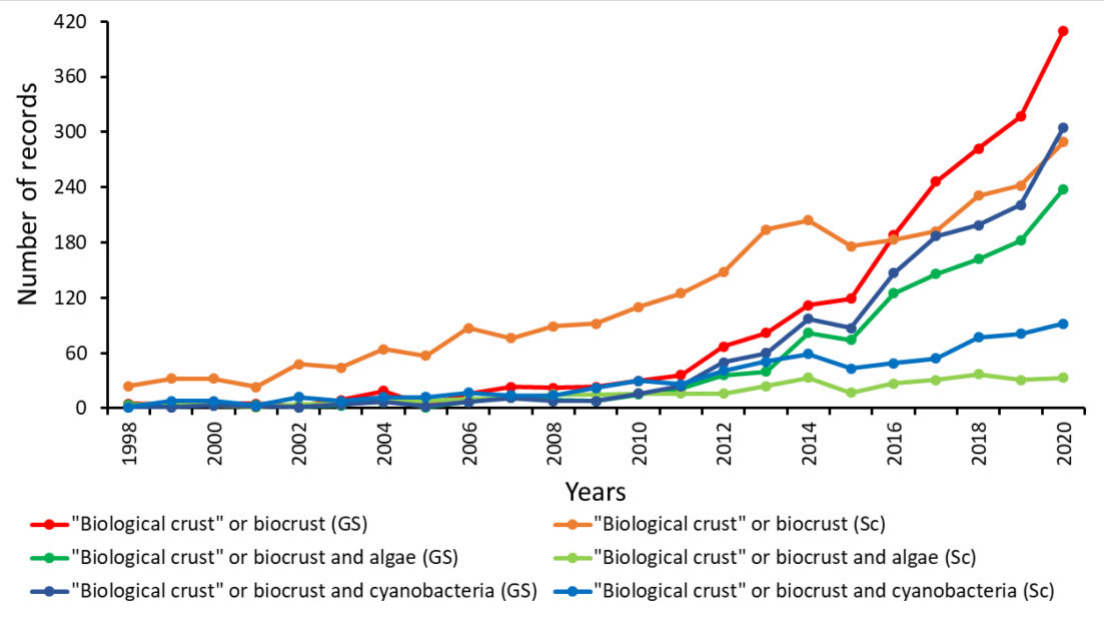
Literature production concerning biocrusts over recent decades based on searches of the terms ‘biocrust’ and ‘biological crust’ and by crossing these terms with ‘algae’ or ‘cyanobacteria’ in a popular (Google Scholar—GS) and a scientific (Scopus—Sc) database. Sources: Google Scholar, https://scholar.google.com; Scopus, http://www.scopus.com/scopus/search/form.urli; accessed November 2021.

Based on our survey, the scientific production focusing on oxygenic photoautotrophic microorganisms in biocrusts has increased, with both algae and cyanobacteria showing consistent increases from 1998 to 2020 for the academic Scopus database and the more accessible Google Scholar database. Two events, the publication of Belnap et al. (2001) and the International Workshop in 2010, seem to have been relevant in expanding interest in biocrusts, possibly contributing to the large increases in the number of articles published and to the exponential increase in interest in this topic. Even with these increases, there was proportionally much lower interest in algae than in cyanobacteria (Fig. 1). It is interesting that although the terms biocrust and biological crusts were coined and widely used in academic works, they have recently become more common and widely found in nonacademic texts. There was an almost constant increase in interest in biocrusts from 2000 to 2020, but focus on oxygenic photoautotrophic microorganisms of this community increased only more recently, just after 2010.

**Figure 2.**
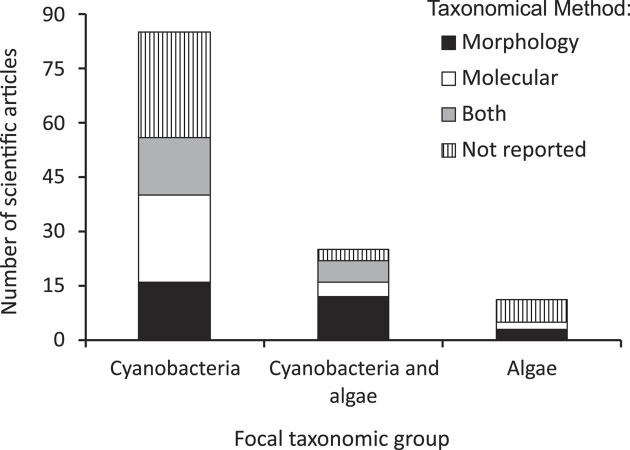
Number of scientific articles focused on biocrusts that cite a species list of cyanobacteria, algae or both.

Although interest in algae and cyanobacteria of biocrusts has been increasing, detailed information at the levels of genus or species is still rare for these oxygenic photoautotrophic microorganisms. Although there have been hundreds of studies on biocrusts that cite algae (1187 in Google Scholar and 369 in Scopus) or cyanobacteria (1448 in Google Scholar and 734 in Scopus) (see Fig. 1), only 121 were found to contain a species list. These studies with more taxonomic details support the much lower interest in algae than in cyanobacteria, since only 36 mentioned the species of algae in samples, while 85 articles listed exclusively species of cyanobacteria (Fig. 2). Figure 2 also presents the results regarding the methods used to identify the species. Many studies (38; 31.4% of our list) provided no information about the identification method used, but we detected some interesting trends based on the remaining scientific articles. The studies exclusively focused on cyanobacteria were mainly based on modern molecular methods (40 studies), with only 16 studies based exclusively in traditional morphology-based methods. The studies focused on algae or in both taxonomic groups were more conservative in terms of the method used to identify the microorganisms, with 15 of them based exclusively on morphology and only 6 based exclusively on molecular biology (Fig. 2).

**Figure 3.**
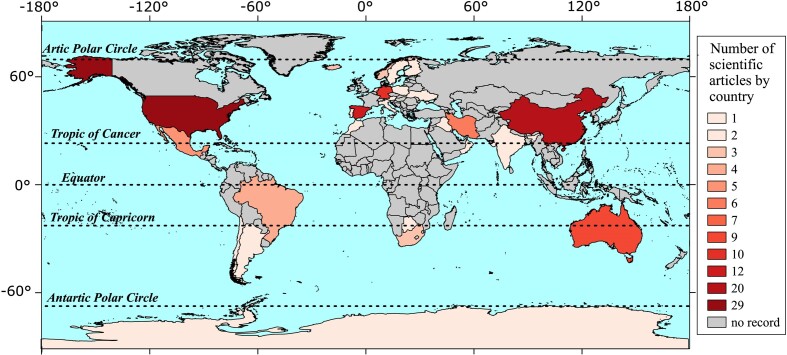
Gradient world map based on the number of articles citing species present in biocrusts by country.

The 121 articles with a species list indicate spatial tendencies, with a clear geographic distribution pattern. They were produced in just 23 countries (and Antarctica) among the more than 200 countries of the world (Fig. 3), and almost all of them were performed in temperate regions, highlighting a lack of knowledge for the tropics (Fig. 3; Table 1). Furthermore, of these 121 scientific articles, 50% resulted from studies performed in USA (29), China (20), and Spain (12). Among the few studies that were developed between the Tropic of Capricorn and the Tropic of Cancer, important contributions came from Australia (nine studies, but only six sites in the tropical region), Brazil (four) and Mexico (four).

**Table 1.** List of the most frequently reported eukaryotic algae (18 species) in scientific articles found in the Scopus database, with the number of articles in which each species is cited, the countries where studies were performed and a pie chart showing the proportion of studies in temperate (blue), tropical (red), and polar (light blue) regions. The list was ordered by region, from temperate to tropical, followed by frequency in papers and finally by alphabetic order.

Algae species (Phyllum)	Papers	Countries	Region 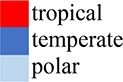
***Diplosphaera chodatii*** (Chlorophyta)	5	Germany (3), Norway, and Chile	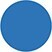
***Elliptochloris subsphaerica*** (Chlorophyta)	5	Germany (3), Chile, and Iceland	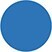
***Stichococcus bacillaris*** (Chlorophyta)	5	Germany (3), Iceland, and Norway	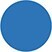
***Myrmecia bisecta*** (Chlorophyta)	4	Chile, Germany, Iceland, and Norway	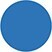
***Tetracystis* sp**. (Chlorophyta)	4	Germany (2), Iceland, and Norway	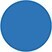
***Chloroidium ellipsoideum*** (Chlorophyta)	4	Germany (3) and Norway	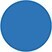
***Klebsormidium* cf. *nitens*** (Charophyta)	3	Chile, Germany, and Iceland	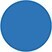
***Nannochloris* sp**. (Chlorophyta)	3	Germany (2) and Iceland	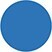
***Bracteacoccus minor*** (Chlorophyta)	3	Germany (3)	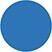
***Bracteacoccus* sp**. (Chlorophyta)	9	USA (3), Germany (2), Chile, Iceland, Norway/Iceland, and Spain	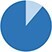
***Chlamydomonas* sp**. (Chlorophyta)	6	Chile, China, Germany, Iceland, Norway, and Norway/Iceland	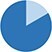
***Chlorococcum* sp**. (Chlorophyta)	6	Germany, Iceland, Israel, Norway, Norway/Iceland, and USA	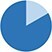
***Diplosphaera* sp**. (Chlorophyta)	4	China, Iceland, Norway/Iceland, and USA	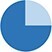
***Klebsormidium flaccidum*** (Charophyta)	4	Antarctica/Arctic, Germany, Iceland, and USA	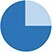
***Klebsormidium* sp**. (Charophyta)	7	Germany (2), USA, Brazil, Chile, Germany/USA, and Norway/Iceland	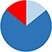
***Stichococcus* sp**. (Chlorophyta)	7	USA (2), Brazil, Chile, Germany, Israel, and Norway/Iceland	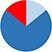
***Chlorella vulgaris*** (Chlorophyta)	7	China, Iceland, Norway, Germany, Mexico, Poland, and USA	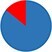
***Chlorella* sp**. (Chlorophyta)	6	Germany (2), Brazil, Chile, Poland, and USA	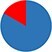

The most frequently found algal taxa (species or genera) for the 26 articles focused on algae and cyanobacteria or only on algae are listed in Table 1, with information on phylum and countries and regions. *Bracteacoccus* sp. was the most commonly cited taxa, being present in nine articles; followed by *Chlorella vulgaris*, and *Klebsormidium* sp. and *Stichococcus* sp. in seven; *Chlorella* sp., *Chlorococcum* sp., and *Chlamydomonas* sp. in six; *Diplosphaera chodatii, Elliptochloris subsphaerica*, and *Stichococcus bacillaris* in five; *Chloroidium ellipsoideum, Diplosphaera* sp., *Myrmecia bisecta, Klebsormidium flaccidum*, and *Tetracystis* sp. in four; and finally, *Bracteacoccus minor, Klebsormidium* cf. *nitens*, and *Nannochloris* sp. in three. Among these 18 most abundant taxa (species and genera), 15 are chlorophytes and three are charophytes. Other taxa were not listed because they were present in only few studies (less than 8% of the studies). The most cited taxa were more all found in temperate regions, with only three, all green algae (Chlorophyta and Charophyta), occurring also in the tropical region (Table 1).

The species most commonly found in the 121 articles focused on cyanobacteria and algae or only on cyanobacteria that cited a list of species present in biocrusts are listed in Table 2. The most frequent taxa in the studies were *Nostoc* sp., reported in 65 articles; *Microcoleus vaginatus* in 46; *Scytonema* sp. in 40; *Leptolyngbya* sp. in 36; *Microcoleus* sp. in 29; *Chroococcidiopsis* sp. in 24; *Tolypothrix* sp. and *Phormidium* sp. in 23; *Microcoleus steenstrupii* in 19; *Oscillatoria* sp. in 18; *Nostoc commune* in 16; *Schizothrix* sp. in 13; *Calothrix* sp. in 12; *Lyngbya* sp. in 11; *Chroococcus* sp. and *Trichocoleus* sp. in 10; *Microcoleus paludosus* and *Scytonema hyalinum* in 9; *Tolypothrix distorta* in 8; and finally, *Anabaena* sp., *Phormidium tenue* (currently *Leptolyngbya tenuis*), and *Synechococcus* sp. in 7. The other species were mentioned very rarely (less than 5% of the studies) and are not included in the table. For taxonomical purpose, it is relevant to mention that *M. steenstrupii* is now considered a complex divided into new genera (*Funiculus, Parifilum, Arizonema, Crassifilum, Crustifilum*, and *Allocoleopsis*) belonging to Coleofasciculaceae (Fernandes et al. 2021, Strunecký et al. 2023). Many species of cyanobacteria were distributed in both temperate and tropical regions, with 20 of the 22 most abundant species being in both regions and only 2 species being exclusively found in temperate regions (Table 2).

**Table 2.** List of the most frequently reported cyanobacteria (22 species) in scientific articles found in the Scopus database, with the number of articles in which each species is cited, countries where studies were performed, and a pie chart showing the proportion of studies in temperate (blue) and tropical (red) regions. Data of Australia are showed as Australia (tropical) or Australia (temperate), depending on study location performed above or below the Tropic of Capricorn, respectively. The list was ordered by region, from temperate to tropical, followed by frequency in papers and finally by alphabetic order.

Cyanobacteria species	Papers	Countries	Region 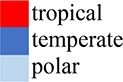
***Trichocoleus* sp**.	10	USA (5), Spain (2), China, Germany, and Iran	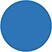
***Phormidium tenue***	7	China (7)	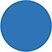
***Nostoc* sp**.	65	USA (19), China (10), Iran (4), Australia (4 tropical; 1 temperate), Israel (5), Germany (3), Brazil (3), Spain (3), South Africa (3), Mexico (2), Argentina, Chile, India, Iceland, Poland, Norway/Iceland, and Portugal	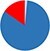
***Leptolyngbya* sp**.	36	USA (9), Australia (3 temperate, 3 tropical), Spain (5), Germany (3), South Africa (3), Brazil (2), Austria, Chile, China, Iceland, Iran, Mexico, Morocco, and Norway/Iceland	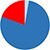
***Microcoleus* sp**.	29	USA (7), Spain (6), China (5), South Africa (3), Australia (1 temperate; 1 tropical), Botswana, Chile, Iceland, India, Israel, and Norway/Iceland	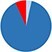
***Phormidium* sp**.	23	Spain (4), South Africa (3), Australia (2 tropical,1 temperate), USA (3), China (2), Botswana, Chile, India, Iran, Iraq, Israel, Mexico, and Norway/Iceland	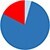
***Oscillatoria* sp**.	18	China (3), Iran (3), USA (3), Australia (1 temperate; 1 tropical), Argentina, Botswana, Brazil, India, Iraq, Israel, and Norway/Iceland	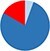
***Microcoleus steenstrupii***	19	USA (10), Spain (4), Israel (2), Botswana, Brazil, and South Africa	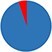
***Microcoleus vaginatus***	46	USA (20), China (5), Israel (4), Iran (3), Spain (3) Brazil (2), Germany (2), Argentina, Australia, Chile, Iceland, Mexico, Poland, and South Africa	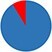
***Scytonema* sp**.	40	USA (16), Australia (5 tropical; 1 temperate), China (4), Israel (4), Spain (3), Botswana, Brazil, Ecuador, India, Iceland, Iran, and South Africa	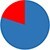
***Chroococcidiopsis* sp**.	24	USA (7), Spain (3), South Africa (3), Brazil (3), China (2), Mexico (2), Australia (temperate), Chile, Iran, and Ukraine	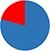
***Tolypothrix* sp**.	23	USA (12), China (2), Iran (2), Spain (2), Australia, Brazil, Israel, Poland, and South Africa	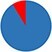
***Nostoc commune***	16	Spain (5), Australia (3 tropical), Argentina, China, Iran, Ecuador, Germany, Mexico, Poland, and USA	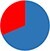
***Schizothrix* sp**.	13	Australia (1 temperate, 1tropical), Israel (2), Spain (2), USA (2), Brazil, India, Iran, Mexico, and Morocco	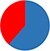
***Calothrix* sp**.	12	USA (4), Brazil (2), Israel (2), Australia, Iran, South Africa, and Spain	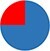
***Lyngbya* sp**.	11	USA (3), China (2), Germany (2), Argentina, India, Iran, and Morocco	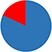
***Chroococcus* sp**.	10	Australia (1temperate, 1tropical), Brazil, India, Iran, Israel, Mexico, Morocco, Poland, and Spain	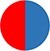
***Microcoleus paludosus***	9	Australia (1 temperate, 2 tropical), USA (2), Botswana, China, Spain, and South Africa	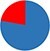
***Scytonema hyalinum***	9	Spain (4), Australia (tropical), Botswana, Brazil, Mexico, and USA	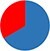
***Tolypothrix distorta***	8	Spain (4), Australia (2 tropical), Mexico, and South Africa	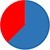
***Anabaena* sp**.	7	Australia (tropical), China, India, Iran, Mexico, Portugal, and USA	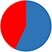
***Synechococcus* sp**.	7	USA (3), Australia (temperate), Botswana, India, and Mexico	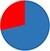

**Figure 4.**
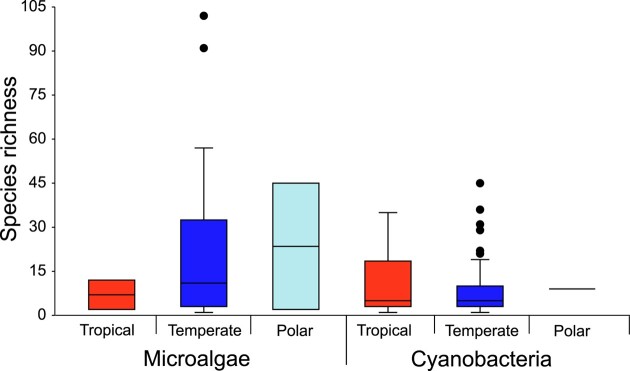
Species richness reported in scientific articles by taxonomic group (microalgae or cyanobacteria) and by region (tropical or temperate). There was no significant difference (*P* > .05, Wilcoxon test) between tropical and temperate regions for the data of cyanobacteria and comparisons were not performed for microalgae, since polar and tropical regions were not sufficiently sampled.

Scarcity of data precluded comparing algae richness by region, although the unique study for the tropics listed 12 species, which was higher than the median (three species) but smaller than the mean (16.8 species) observed in temperate regions (Fig. 4). For cyanobacteria there were 22 articles for tropical regions and 88 for temperate regions, which allowed us to do statistical comparisons that revealed no significant difference (Kruskal–Walis, Chi^2^ = 1.79, *P*-value = .177) between the richness of tropical (median = 5.0; mean = 10.8) and temperate regions (median = 5.0; mean = 7.7) (Fig. 4). For algae, we found 26 articles for temperate regions and only two articles for tropical and for polar regions. Even with this limitation, we performed the same statistical analysis and comparisons revealed no significant difference (Kruskal–Walis, Chi^2^ = 0.56, *P*-value = .754) between the richness of tropical (median = 7.0; mean = 7.0), temperate (median = 11.0; mean = 21.8) and polar regions (median = 23.5; mean = 23.5) (Fig. 4).

In relation to the environment where each of the most common species was observed, almost all papers described the local climate and substrate where the biocrusts samples were taken (Supplementary Material - S2 and S3). Climate was not always clearly mentioned or adjusted to an international system. An example is environments mentioned as ‘extreme’ by authors, without more detail about climate classification. In some cases, the authors did not mention the climate type, but it was possible to infer through the description of local conditions described in other studies carried out in the same geographic coordinates. In the temperate region, the survey detected 13 taxa in rocky environments and 24 in the soil, while only soils were studied in the tropical region, with 18 taxa recorded. Thus, the epilithic biofilms (biofilms growing on rock surfaces) were only reported for temperate regions and they were exclusively focused on cyanobacteria, while studies performed in biocrusts samples from soil were more common and focused on cyanobacteria and eukaryotic algae. The most studied environments usually coincided with taxonomical lists showing higher species richness.

## Discussion

The results of the present study show increasing scientific interest in biocrusts, which is probably a consequence of recent studies exploring the important ecological services they provide, such as those related to water retention and fundamental roles in N and C fixation (Sepehr et al. 2019). Recent studies have focused on potential management of this community for environmental restoration (Rodríguez-Caballero et al. 2018b, Blankenship et al. 2019, Antoninka et al. 2020), which may be more attractive than more basic studies focused on taxonomic surveys. However, taxonomic, life history and biogeographic knowledge are still fundamental to a more specific understanding of this little understood community and would benefit researchers initiating studies in a specific region. Unfortunately, the information available in scientific articles reveals that there is little or no knowledge for many regions and that the taxonomy of oxygenic photoautotrophic microorganisms, mainly eukaryotes, is still poorly explored for biocrusts. This lack of knowledge is also very relevant when considering that oxygenic photoautotrophic organisms of biocrusts can be threatened by anthropogenic impacts, such as trampling (Yang et al. 2020), burning (Aanderud et al. 2019), and climate change (Ferrenberg et al. 2015, Steven et al. 2015).

The larger number of studies focused on cyanobacteria than on eukaryotic algae in biocrusts is due to the very important ecological roles of these prokaryotic organisms, such as their abilities to fix C and N, provide soil resistance to erosion and contribute to soil moisture retention (Zhao et al. 2010, Steven et al. 2012, Cantón et al. 2020). These processes cause relevant changes to the soil that favor the establishment of other organisms in biocrusts and are essential to ensure subsequent successional changes and the establishment of more complex vegetation in many systems (Zhang et al. 2016). However, even though eukaryotic algae are not able to fix N, this is not a justification for not performing additional studies focused on these organisms in biocrusts. Algae also play extremely important roles for the establishment and development of biocrusts, being fundamental to soil stability (Sepehr et al. 2019), and contributing to the generation of conditions that will also determine successional changes (Hu et al. 2002). For example, many eukaryotic algae of biocrusts have special mechanisms for growth in sites with high salinity and drought conditions. Some desiccation tolerant algae will release exopolymeric substances (EPS) that prevent water loss and contribute to the formation of biofilms by aggregation of cells and colonies (Kumar et al. 2018). The strategies adopted by algae to resist stressful conditions affect ecosystem functioning. Both EPS and the above-mentioned aggregates, e.g. can contribute to soil water retention, as observed for cyanobacteria of biocrusts (Mazor et al. 1996, Adessi et al. 2018). Another mechanism is the production of organic osmolytes, which is usually performed by halotolerant algae (Shetty et al. 2019). In addition to the effects of cyanobacteria, the changes that eukaryotic algae cause to the environment have the potential to restore degraded areas (Condon et al. 2019). Sommer et al. (2020), e.g. evaluated the restoration of an area degraded by piles of potassium tailings through the development of artificial biocrusts. These researchers found that algae contributed to reducing the erosion caused by desiccation by providing greater water availability and soil particle aggregation. Colonization by biofilms is generally the first step in species succession and will, depending on the region, increase the structural complexity of the system so that bryophytes and vascular plants are able to grow (Ciccazzo et al. 2016, Read et al. 2016, Rubio and Lázaro 2023).

The great heterogeneity in the geographic distribution of studies focused on biocrusts may be a consequence of their geographic distribution (see Rodriguez-Caballero et al. 2018a), or due to economic differences among countries in temperate and tropical regions, resulting in different levels of research investments. Temperate countries are generally wealthier, as shown by their higher national per capita income compared to tropical countries (Gallup et al. 2000, Sachs 2001). The consequence is that a vast area has not been sampled or studied, and so it cannot be affirmed that this is a result of the absence of important biocrust communities. Although much more is known about oxygenic photoautotrophic microorganisms of biocrusts of temperate regions, it is possible that biocrust species diversity is higher in the tropics and that important metabolic or ecological information may be being lost or neglected in this region. Machado-de-Lima et al. (2019) performed a study focused on cyanobacteria in biocrusts of the Brazilian Savanna and detected a diverse community, although lacking many species typically found in temperate biocrusts. According to the concept of latitudinal diversity gradient, species diversity of terrestrial ecosystems tends to increase from polar to tropical regions (Pianka 1966, Willig et al. 2003). This has been observed for many taxonomic groups and communities and may also occur with biocrusts, but there is little literature available to evaluate geographic patterns. The species richness data presented here show that tropical biocrusts could indeed have biodiversity levels at least similar to those of temperate biocrusts. In an interesting study, Rodriguez-Caballero et al. (2018a) applied environmental niche modeling to create a global relative suitability map showing current biocrust distribution patterns. The result suggested that biocrusts are indeed more commonly observed in temperate regions, but they also cover very extensive tropical areas in Latin America, Sahel, South and East Africa, Middle East, India, and a large portion of Australia. Thus, more studies are necessary to clarify whether the higher diversity of oxygenic photoautotrophs in biocrusts of temperate regions is a natural geographic pattern or if it is a consequence of a lack of studies in the tropics. The latter scenario is probably due to different investments in research per region and not from true differences in the ecological services played by biocrusts. Further, since the latitudinal gradient of diversity describes a general, but not absolute, pattern for all groups of species, spatial trends for biocrusts remain completely unclear.

The high concentration of studies in some countries is also reflected in species reports, with 20 of the 22 species of cyanobacteria most-commonly cited in scientific studies being mentioned in studies in USA (total of 19 species) or China (total of 13 species). These trends reinforce the probable influence of the availability of funding for scientific research in these countries, which may be introducing bias that obscures real trends present in nature. Even considering this probable bias, some taxa are certainly observed more frequently and are more cosmopolitan on a global scale. For example, the eukaryotic algae *Klebsormidium* (Charophyta) and *Chlorella* were reported in six and four studies, respectively, with both taxa being identified in tropical and temperate biocrusts. For cyanobacteria, the higher number of studies resulted in greater frequencies, with *Nostoc, Microcoleus* (including *M. vaginatus*), *Scytonema*, and *Leptolyngbya* being reported in 51, 64, 31, and 25 studies, respectively, from tropical and temperate regions. Some of these taxa are considered very common or even cosmopolitan in biocrusts, such as *M. vaginatus, Nostoc*, and *Scytonema* (Büdel 2003, Samolov et al. 2020). Machado-de-Lima et al. (2019) warned that many species that are commonly recorded in temperate ecosystems were not detected in their study in a tropical region, suggesting that relevant differences may occur and should be considered by future research.

In relation to the characteristics of the environments where biocrusts are found, our data compilation shows occurrences from extremely dry to highly humid environments (Castillo-Monroy et al. 2016, Navas Romero et al. 2019, Hakkoum et al. 2020, Samolov et al. 2020). However, information on environmental characteristics and species occurrence seems to be limited, since there are more studies in arid and semiarid climate, and almost all genera were reported for these regions (Román et al. 2018, Williams et al. 2018, Roncero-Ramos et al. 2019, Szyja et al. 2019, Romero et al. 2020, Zhao et al. 2021). Although these records could generate a perception that biocrusts have more important roles in dry areas, where they possibly have a greater representativeness in the local autotrophic biomass, it is also possible that the greater number of studies in these regions are generating a very incomplete view and more studies should be developed in humid areas to try better identify their ecological functions in these environments. The species list showed that the most common cyanobacteria genera (*Nostoc, Microcoleus, Schizothrix*, and *Scytonema*) and eukaryotic algae (*Bracteacoccus, Chlamydomonas, Chlorella, Diplosphaera*, and *Klebsormidium*) showed no clear pattern of occurrence related to specific environments and were recorded in almost all kinds of climate or growing substrate, although there is a lack of information for epilithic biofilms in the tropics. Finally, none of the most common taxa were observed to be restrict or specific to a particular environment, showing that most species or genera are probably cosmopolitan. We believe that a high number of cosmopolitan species makes the taxonomic identification more difficult, since there are a high number of possibilities to find one among a higher number of species. Species that are endemic or typical of a region would be more easily identified as they are expected to be found in samples from this location. Thus, a review of literature like the present study is necessary to facilitate taxonomical and ecological studies. Regarding the substrate approach, it is important to understand why rock systems are much less studied (13 studies) than soils (42 studies), as also observed by Weber et al. (2022). Perhaps this is due to the smaller coverage area of these systems or because there is greater interest in soils due to more potential uses by humans. Although there is greater knowledge about the functions of biocrusts in soils, such as soil nutrient enrichment, mitigation of erosion, moisture maintenance, control of water infiltration, and soil development (Beymer and Klopatek 1991, Belnap and Gillette 1997, Belnap et al. 2001, Xiao et al. 2018), these biological communities can also be very relevant in rocky environments devoid of soil (Abrantes et al. 2023).

Standardization in the methods used to identify species must also be considered, as it differed greatly between the groups of organisms studied. Cyanobacteria are mainly identified by molecular methods, while algae identification remains based on morphologically based taxonomy. Molecular biology techniques allow us to increase accuracy and speed up the species identification process (Manoylov 2014). If this trend continues, it is possible that studies focusing on cyanobacteria will advance at a much faster pace than those focusing on eukaryotic algae, increasing the proportional deficit in knowledge about the real importance of these microorganisms in biocrusts.

In summary, the survey of the literature showed that there is increasing interest in biocrusts due to the ecological services they provide. These services are based essentially on the facilitation model in successional progress, in which species from one stage of succession modify habitat characteristics in a way that facilitates the establishment of species typical of more advanced stages (Connell and Slatyer 1977). There is great interest in this process because it could be managed for environment restoration. The interest in biocrusts should be even higher because they are under various threats, mainly related to burning events and trampling (Abrantes et al. 2023), that can diminish or eliminate their ecological roles. Even though they are essential throughout the world, studies on biocrusts have a highly heterogeneous geographic distribution, with knowledge of this community lacking for many ecosystems, particularly those of the tropics. Although it is not very clear, these geographic gaps may be due to a latitudinal pattern of investment in research, as countries in temperate regions are generally richer than countries located in tropical regions. However, since the relationship with GDP was not evident, there are probably other reasons such as scientific workforce, potential research lines established in the field, or ecological aspects, which must be better understood to better direct efforts in studies of this community on a global level. Taxonomic heterogeneity was also detected for oxygenic photoautotrophic microorganisms in biocrusts, with almost all studies being focused on cyanobacteria due to their ability to fix atmospheric N. Few studies have focused on eukaryotic algae, although they also play many important roles in soil stabilization and species succession. It is probable that this taxonomic heterogeneity influences, and is influenced by, the geographic heterogeneity of studies. Even with limited information, we provide here a large list of cyanobacteria and algae taxa that have been found in biocrusts (Table S1) and highlight the species most commonly found in studies by region. This represents a taxonomic list that will be useful for the development of new studies by providing researchers with some support on which taxa they would likely find in their samples, although new records should also be frequent due to the small number of studies in general. We expect that the data and list we assembled, based on information, i.e. dispersed in the literature, to support more detailed studies focused on cyanobacteria and algae in biocrusts and contribute to diminishing gaps in scientific production on oxygenic photoautotrophic microorganisms in biocrusts.

## Supplementary Material

fiae086_Supplemental_Files
