# Supplementary material for: Organizing a global list of cyanobacteria and algae from soil biocrusts evidenced great geographic and taxonomic gaps
Source: FEMS Microbiol Ecol. 2024 May 30;100(7):fiae086. doi: 10.1093/femsec/fiae086 (PMC11221558; doi:10.1093/femsec/fiae086)
Supplement: fiae086_Supplemental_Files [file fiae086_supplemental_files.zip › Supplementary Material S1.docx]

**Supplementary Material - S1**

**Taxonomic information present in all papers obtained, mentioning authors, year of publication, species identification method, species present, country where the study was performed. Complete references are listed after the table.**

| Paper 1 – Aanderud ZT, Bahr J, Robinson DM *et al.* (2019) | |
| --- | --- |
| Country: United States (USA) Taxonomical Method(s): Morphology and Molecular Biology | |
| **Algae:** | **Cyanobacteria:**  *Chroococcidiopsis*  *Microcoleus vaginatus*  *Nostoc* |
| Paper 2 – Abed RMM, Tamm A, Hassenrück C *et al.* (2019) | |
| Country: Oman Taxonomical Method(s): Molecular Biology | |
| **Algae:** | **Cyanobacteria:**  Coleofasciculaceae sp.  *Dactylococcopsis*  Nostocales sp.  Oxyphotobacteria  *Tychonema* |
| Paper 3 – Antoninka A, Bowker MA, Chuckran P *et al.* (2018) | |
| Country: United States (USA) Taxonomical Method(s): not reported | |
| **Algae:** | **Cyanobacteria:**  *Microcoleus vaginatus*  *Nostoc* sp.  *Scytonema* sp. |
| Paper 4 – Antoninka A, Bowker MA, Reed SC *et al.* (2016) | |
| Country: United States (USA) Taxonomical Method(s): not reported | |
| **Algae:** | **Cyanobacteria:**  *Microcoleus* sp.  *Nostoc* sp.  *Scytonema* sp. |
| Paper 5 – Arp G, Bissett A, Brinkmann N, *et al.* (2010) | |
| Country: Germany Taxonomical Method(s): Morphology and Molecular Biology | |
| **Algae:**  *Achnanthidium minutissimum*  *Achnanthidium* sp.  *Amphora pediculus*  cf. *Amphora*  *Gomphonema* sp.  *Navicula* sp.  *Navicula tripunctata*  *Navicula veneta*  *Nitzschia palea*  *Pinnularia* sp.  *Surirella brebissonii* | **Cyanobacteria:**  *Aphanothece castagnei*  *Chamaesiphon* sp.  *Hyella fontana*  *Leptolyngbya foveolarum*  *Lyngbya* sp.  *Phormidium calcareum*  *Phormidium incrustatum*  *Pseudanabaena* sp.  *Schizothrix calcicola*  *Schizothrix fasciculata*  *Schizothrix pulvinata*  *Tychonema bourrellyi*  *Tychonema* sp.  *Tychonema tenue* |

| Paper 6 – Ayuso SV, Giraldo-Silva A, Barger NN *et al.* (2020) | |
| --- | --- |
| Country: United States (USA) Taxonomical Method(s): Molecular Biology | |
| **Algae:** | **Cyanobacteria:**  *Calothrix* sp.  *Chroococcidiopsis* sp.  *Crinalium* sp.  *Leptolyngbya* sp.  *Microcoleus steenstrupii*  *Microcoleus vaginatus*  *Nostoc* sp.  *Oscillatoria* sp.  *Scytonema* sp.  *Tolypothrix* sp.  *Trichocoleus* sp. |

| Paper 7 – Ayuso SV, Giraldo Silva A, Nelson C *et al.* (2017) | |
| --- | --- |
| Country: United States (USA) Taxonomical Method(s): Molecular Biology | |
| **Algae:** | **Cyanobacteria:**  *Microcoleus vaginatus*  *Microcoleus steenstrupii*  *Nostoc* sp. |

| Paper 8 – Bailet B, Bouchez A, Franc A, *et al.* (2019) | |
| --- | --- |
| Country: Sweden, Finland, Norway and Iceland Taxonomical Method(s): Morphology and Molecular Biology | |
| **Algae:**  *Achnanthidium*  *Achnanthidium minutissimum*  *Amphora pediculus*  *Aulacoseira ambigua*  *Caloneis*  *Cocconeis placentula*  *Cyclotella meneghiniana*  *Encyonema neogracile*  *Entomoneis*  *Eolimna minima*  *Eunotia*  *Eunotia incisa*  *Eunotia minor*  *Fragilaria*  *Fragilaria capucina*  *Fragilaria gracilis* (Currently *Fragilaria radians*)  *Gomphonema*  *Halamphora oligotraphenta*  *Halamphora veneta*  *Navicula*  *Nitzschia*  *Nitzschia dissipata*  *Nitzschia palea*  *Rhoicosphenia abbreviata*  *Staurosira pinnata* (Currently *Staurosirella leptostauron*)  *Tabellaria flocculosa*  *Ulnaria*  *Ulnaria ulna* | **Cyanobacteria:** |

| Paper 9 – Bao T, Zhao Y, Gao L *et al.* (2019) | |
| --- | --- |
| Country: China Taxonomical Method(s): Molecular Biology | |
| **Algae:** | **Cyanobacteria:**  *Nostoc* sp.  *Phormidium calcicola*  *Phormidium tenue* (Currently *Leptolyngbya tenuis*) |
| Paper 10 – Bao T, Zhao Y, Yang X *et al.* (2019) | |
| Country: China Taxonomical Method(s): Molecular Biology | |
| **Algae:** | **Cyanobacteria:**  *Nostoc* spp.  *Phormidium calciola*  *Phormidium tenue* (Currently *Leptolyngbya tenuis*) |
| Paper 11 – Bastida F, Jehmlich N, Ondoño S *et al.* (2014) | |
| Country: Spain Taxonomical Method(s): Molecular Biology | |
| **Algae:** | **Cyanobacteria:**  Chroococcales  Gloeobacterale*s*  *Microcoleus vaginatus*  Nostocales  Oscillatoriales  Prochlorales  *Prochlorothrix hollandica* |
| Paper 12 – Becerra-Absalón I, Johansen JR, Muñoz-Martín MA *et al.* (2018) | |
| Country: Mexico Taxonomical Method(s): Morphology and Molecular Biology | |
| **Algae:** | **Cyanobacteria:**  *Chroakolemma edaphica* (Currently *Timaviella edaphica*)  *Chroakolemma opaca*  *Chroakolemma pellucida*  *Chroakolemma* spp. |

| Paper 13 – Becerra-Absalón I, Muñoz-Martín MÁ, Montejano G *et al.* (2019) | |
| --- | --- |
| Country: Mexico Taxonomical Method(s): Morphology and Molecular Biology | |
| **Algae:** | **Cyanobacteria:**  *Calothrix parietina*  *Chroakolemma opaca*  *Chroakolemma pellucida*  *Chroococcidiopsis* sp.  *Kamptonema* sp.  *Leptolyngbya frigida* (currently *Stenomitos frigidus*)  *Leptolyngbya* sp.  *Microcoleus vaginatus*  *Nostoc commune*  *Nostoc* sp.  *Oculatella atacamensis*  *Oculatella* sp.  *Porphyrosiphon notarisii*  *Schizothrix* sp.  *Scytonema crispum* (currently *Heterosytonema crispum*)  *Scytonema hyalinum*  *Synechococcus* sp.  *Tolypothrix distorta* |
| Paper 14 – Belnap J, Büdel B (2016) | |
| Country: Germany and United States (USA) Taxonomical Method(s): Not reported | |
| **Algae:**  *Bracteacoccus giganteus*  *Klebsormidium* sp.  *Neochlorosarcina negevensis* | **Cyanobacteria:** |
| Paper 15 – Bengtsson MM, Wagner K, Schwab C, *et al.* (2018) | |
| Country: Austria Taxonomical Method(s): Molecular Biology | |
| **Algae:**  *Achnanthidium minutissimum* | **Cyanobacteria:**  *Leptolyngbya* |

| Paper 16 – Borchhardt N, Baum C, Thiem D *et al.* (2019) | |
| --- | --- |
| Country: Norway Taxonomical Method(s): Morphology | |
| **Algae:**  *Characiopsis minuta*  *Chlamydomonas* sp.  *Chlorella vulgaris*  *Chlorococcum* sp.  *Chloroidium ellipsoideum*  *Chloromonas* cf. *palmelloides* (Currently *Chloromonas reticulata*)  *Coccomyxa* cf. *confluens*  *Coccomyxa curvata* (Currently *Neocystis curvata*)  *Coccomyxa gloeobotrydiformis* (Currently *Coenobotrys gloeobotrydiformis*)  *Coccomyxa simplex* (Currently *Pseudococcomyxa simplex*)  *Coelastrella aeroterrestrica*  *Coelastrella rubescens*  *Coenochloris* sp.  *Desmodesmus abundans*  *Diplosphaera chodatii*  *Elliptochloris bilobata*  *Eustigmatos vischeri* (Currently *Vischeria vischeri*)  *Fasciculochloris boldii*  *Gloeocystis* sp.  *Graesiella emersonii*  *Interfilum* sp.  *Klebsormidium* cf. *crenulatum*  *Klebsormidium* cf. *dissectum*  *Leptosira obovata*  *Macrochloris cohaerens*  *Muriella terrestris*  *Mychonastes homo*sp*haera*  *Myrmecia bisecta*  *Neocystis brevis*  *Neocystis* cf. *brevis*  *Neocystis* sp.  *Pleurogaster lunaris*  *Pseudodictyochloris multinucleata*  Sp*orotetras polydermatica*  *Stichococcus bacillaris*  *Stichococcus* cf. *allas* (Currently *Deuterostichococcus tetrallantoideus*)  *Tetracystis* sp. | **Cyanobacteria:** |
| Paper 17 – Bowker MA, Antoninka AJ, Chuckran PF (2020) | |
| Country: United States (USA) Taxonomical Method(s): Not reported | |
| **Algae:** | **Cyanobacteria:**  *Microcoleus*  *Nostoc*  *Scytonema* |

| Paper 18 – Büdel B, Williams WJ, Reichenberger H (2018) | |
| --- | --- |
| Country: Australia Taxonomical Method(s): Morphology | |
| **Algae:** | **Cyanobacteria:**  *Nostoc commune*  *Scytonema* sp.  *Symploca* sp.  *Symplocastrum purpurascens* |
| Paper 19 – Caesar J, Tamm A, Ruckteschler N *et al.* (2018) | |
| Country: Germany Taxonomical Method(s): Not reported | |
| **Algae:**  *Klebsormidium* sp. | **Cyanobacteria:** |
| Paper 20 – Cano-Díaz C, Mateo P, Muñoz-Martín MA *et al.* (2018) | |
| Country: Spain Taxonomical Method(s): Morphology and Molecular Biology | |
| **Algae:** | **Cyanobacteria:**  *Chroococcus* sp.  *Leptolyngbya* sp.  *Microcoleus* sp.  *Mojavia* sp.  *Nodosilinea* sp.  *Nostoc* sp.  *Roholtiella edaphica*  *Scytonema* sp.  *Spirirestris* sp.  *Trichocoleus* sp. |
| Paper 21 – Cantón Y, Chamizo S, Rodriguez-Caballero E *et al.* (2020) | |
| Country: Spain Taxonomical Method(s): Not reported | |
| **Algae:** | **Cyanobacteria:**  *Chroococcidiopsis* sp.  *Leptolyngbya* sp.  *Leptolyngbya frigida* (currently *Stenomitos frigidus*)  *Microcoleus* sp.  *Microcoleus steenstrupii*  *Nostoc calcicola*  *Nostoc commune*  *Oculatella kazantipica*  *Phormidium* sp.  *Schizothrix* cf. *calcicola*  *Scytonema hyalinum*  *Tolypothrix distorta*  *Trichocoleus desertorum* |
| Paper 22 – Castillo-Monroy AP, Benítez A, Reyes-Bueno F *et al.* (2016) | |
| Country: Ecuador Taxonomical Method(s): Morphology | |
| **Algae:** | **Cyanobacteria:**  *Nostoc commune*  *Scytonema* sp. |

| Paper 23 – Chamizo S, Adessi A, Certini G *et al.* (2020) | |
| --- | --- |
| Country: Italy and Portugal Taxonomical Method(s): Not reported | |
| **Algae:** | **Cyanobacteria:**  *Phormidium ambiguum*  *Scytonema javanicum* |
| Paper 24 – Chamizo S, Adessi A, Torzillo G *et al.* (2020) | |
| Country: Spain Taxonomical Method(s): Not reported | |
| **Algae:** | **Cyanobacteria:**  *Nostoc commune*  *Phormidium ambiguum*  *Scytonema javanicum* |
| Paper 25 – Chamizo S, Mugnai G, Rossi F *et al.* (2018) | |
| Country: Spain Taxonomical Method(s): Not reported | |
| **Algae:** | **Cyanobacteria:**  *Phormidium ambiguum*  *Scytonema javanicum* |
| Paper 26 – Chilton AM, Neilan BA, Eldridge DJ (2018) | |
| Country: Australia Taxonomical Method(s): Molecular Biology | |
| **Algae:** | **Cyanobacteria:**  *Aphanizomenon*  *Cylindrospermum*  *Leptolyngbya*  *Phormidium*  *Tolypothrix*  *Toxopsis* |
| Paper 27 – Chua M, Erickson TE, Merritt DJ *et al.* (2020) | |
| Country: Australia Taxonomical Method(s): Not reported | |
| **Algae:** | **Cyanobacteria:**  *Leptolyngbya* sp.  *Microcoleus* sp.  *Nostoc* sp.  *Scytonema* sp. |
| Paper 28 – Condon LA, Pietrasiak N, Rosentreter (2020) | |
| Country: United States (USA) Taxonomical Method(s): Morphology | |
| **Algae:**  *Bracteacoccus* sp.  *Chlorella* sp.  *Chlorococcum* sp.  *Chlorosarcinopsis* sp.  *Diplosphaera* sp.  *Hantzschia* cf. *amphioxys*  *Heterochlamydomonas* sp.  *Klebsormidium* sp.  *Luticola* sp.  *Macrochloris* sp.  *Micractinium* sp.  *Pinnularia* sp.  *Stichococcus* sp. | **Cyanobacteria:**  *Crinalium* sp.  *Hassallia* sp.  *Hormoscilla* sp.  *Microcoleus* sp.  *Microcoleus steenstrupii*  *Microcoleus vaginatus*  *Nostoc* sp.  *Phormidium* sp.  *Symplocastrum* sp.  *Synechococcus* sp.  *Trichocoleus* sp. |

| Paper 29 – Couradeau E, Giraldo-Silva A, De Martini F *et al.* (2019) | |
| --- | --- |
| Country: United States (USA) Taxonomical Method(s): Morphology and Molecular Biology | |
| **Algae:** | **Cyanobacteria:**  Acaryochlorales  Chroococcales  *Microcoleus vaginatus*  Nostocales  Pseudanabaenales |
| Paper 30 – Couradeau E, Karaoz U, Lim H *et al.* (2016) | |
| Country: United States (USA) Taxonomical Method(s): Molecular Biology | |
| **Algae:** | **Cyanobacteria:**  *Microcoleus steenstrupii*  *Microcoleus vaginatus*  *Nostoc* sp.  *Scytonema* sp.  *Tolypothrix* sp. |
| Paper 31 – Dettweiler-Robinson E, Sinsabaugh RL, Rudgers JA (2018) | |
| Country: United States (USA) Taxonomical Method(s): Not reported | |
| **Algae:** | **Cyanobacteria:**  *Microcoleus* sp.  *Nostoc* sp.  *Scytonema* sp. |
| Paper 32 – Doherty KD, Bowker MA, Antoninka AJ *et al.* (2018) | |
| Country: United States (USA) Taxonomical Method(s): Morphology | |
| **Algae:** | **Cyanobacteria:**  *Microcoleus* sp.  *Nostoc* sp.  *Scytonema* sp. |

| Paper 33 – Dojani S, Kauff F, Weber B *et al.* (2014) | |
| --- | --- |
| Country: South Africa Taxonomical Method(s): Morphology and Molecular Biology | |
| **Algae:** | **Cyanobacteria:**  *Calothrix* sp.  *Chroococcidiopsis* sp.  *Hormoscilla pringsheimii*  *Leptolyngbya* cf. *compacta*  *Leptolyngbya schmidlei*  *Leptolyngbya* sp.  *Leptolyngbya subtilissima*  *Lyngbya* cf. *semiplena*  *Microcoleus chthonoplastes* (currently *Coleofasciculus chthonoplastes*)  *Microcoleus paludosus*  *Microcoleus* sp.  *Microcoleus steenstrupii*  *Microcoleus vaginatus*  *Nostoc* cf. *calcicola*  *Nostoc* cf. *punctiforme*  *Nostoc* sp.  *Oscillatoria limosa*  *Oscillatoria subbrevis*  *Oscillatoria tenuis*  *Phormidium* cf. *aerugineo-caeruleum* (Currently *Potamolinea aerugineocaerulea*)  *Phormidium* cf. *caerulescens Phormidium* cf. *nigrum*  *Phormidium murrayi* (Currently *Wilmottia murrayi*)  *Phormidium* sp.  *Phormidium vulgare* (Currently *Microcoleus vulgaris*)  *Pseudanabaena* cf. *frigida* (Currently *Stenomitos frigidus*)  *Pseudanabaena* cf. *starmachii Pseudanabaena* cf. *tenuis Pseudanabaena minima*  *Pseudanabaena* sp.  *Pseudophormidium hollerbachianum Schizothrix* cf. *arenaria*  *Schizothrix lardacea*  *Scytonema* cf. *millei*  *Scytonema hofmanni*  *Scytonema ocellatum*  *Scytonema* sp.  *Stigonema ocellatum*  *Symplocastrum* cf. *friesii*  *Tolypothrix bouteillei* (Currently *Hassallia bouteillei*)  *Tolypothrix distorta*  *Tolypothrix* sp.  *Trichocoleus* cf. *cavanillesi*  *Trichocoleus* cf. *delicatulus*  *Trichocoleus sociatus* (Currently *Funiculus sociatus*) |

| Paper 34 – Dulić T, Meriluoto J, Palanački MT *et al.* (2017) | |
| --- | --- |
| Country: Iran Taxonomical Method(s): Morphology | |
| **Algae:** | **Cyanobacteria:**  *Aphanocapsa*  *Aphanothece*  *Calothrix*  *Chroococcidiopsis*  *Chroococcus*  *Cyanosarcina*  *Hassallia*  *Homeothrix*  *Microcoleus vaginatus*  *Nostoc*  *Oculatella*  *Schizothrix*  *Scytonema*  *Tolypothrix*  *Trichocoleus* |
| Paper 35 – Fattahi SM, Soroush A, Huang N *et al.* (2020) | |
| Country: Iran Taxonomical Method(s): Not reported | |
| **Algae:** | **Cyanobacteria:**  *Microcoleus vaginatus*  *Nostoc punctiforme* |
| Paper 36 – Fattahi SM, Soroush A, Huang N (2020) | |
| Country: China Taxonomical Method(s): Morphology | |
| **Algae:** | **Cyanobacteria:**  *Microcoleus vaginatus*  *Nostoc punctiforme* |
| Paper 37 – Fernandes VMC, Machado de Lima NM, Roush D *et al.* (2018) | |
| Country: United States (USA) Taxonomical Method(s): Molecular Biology | |
| **Algae:** | **Cyanobacteria:**  *Chroococcidiopsis*  *Leptolyngbya*  *Microcoleus vaginatus*  *Microcoleus steenstrupii*  *Nostoc*  *Phormidium*  *Scytonema*  *Tolypothrix*  *Trichocoleus* |
| Paper 38 – Fischer T (2019) | |
| Country: Germany Taxonomical Method(s): Not reported | |
| **Algae:**  *Zygogonium ericetorum* | **Cyanobacteria:** |

| Paper 39 – Flechtner VR, Johansen JR, Belnap J. (2008) | |
| --- | --- |
| Country: United States (USA) Taxonomical Method(s): Morphology and Molecular Biology | |
| **Algae:**  *Achnanthes coarctata*  *Bracteacoccus* sp.  *Chlorella mirabilis* (Currently *Edaphochlorella mirabilis*)  *Chlorella vulgaris*  *Coccobotrys verrucariae* (Currently *Uvulifera* *verrucariae*)  *Eustigmatos magnus* (Currently *Vischeria magna*)  *Hantzschia abundans*  *Hantzschia amphioxys*  *Heterococcus pleurococcoides*  *Klebsormidium flaccidum*  *Luticola* cf. *dismutica*  *Luticola mutica*  *Luticola nivalis*  *Muelleria* cf. *gibbula*  *Myrmecia astigmatica*  *Myrmecia biatorellae*  *Palmellopsis californica*  *Pinnularia* sp.  *Pseudotetracystis compactis*  *Stichococcus chlorelloides*  *Trebouxia* cf. *aggregata* | **Cyanobacteria:**  *Aphanocapsa fuscolutea*  *Aphanothece maritima*  *Chlorogloeopsis fritschii*  *Chroococcidiopsis edaphica*  *Chroococcus cohaerens*  *Cyanosarcina atroveneta*  *Gloeocapsa biformis*  *Gloeocapsa compacta*  *Hassallia californica*  *Hassallia pseudoramosissima*  *Leibleinia edaphica*  *Leptolyngbya* cf. *crispata*  *Leptolyngbya* cf. *foveolarum*  *Leptolyngbya nostocorum*  *Leptolyngbya* sp.  *Microcheate terrestris*  *Microcoleus vaginatus*  *Nostoc* cf. *bornetii*  *Nostoc desertorum*  *Nostoc paludosum*  *Nostoc punctiforme*  *Nostoc sphaericum*  *Plectonema* cf. *tomasinianum*  *Scytonema obscurum*  *Scytonema ocellatum*  *Synechocystis pevalekii*  *Tolypothrix* cf. *rupestris*  *Tolypothrix distorta* var. *symplocoides*  *Trichocoleus* cf. *delicatulus*  *Trichormus variabilis* |
| Paper 40 – Forster R, Creach V, Sabbe K, *et al.* (2006) | |
| Country: Netherlands Tanonomical Method(s): Morphology and Molecular Biology | |
| **Algae:**  *Amphora* cf. *exigua*  *Amphora laevis* var. *laevissima* (Currently *Amphora* *laevissima*)  *Gyrosigma* sp.  *Navicula arenaria* var. *rostellata*  *Navicula* cf. *mollis*  *Navicula flanatica*  *Navicula gregaria*  *Navicula microdigitoradiata*  *Navicula perminuta*  *Navicula phyllepta*  *Navicula* sp.  *Petrodictyon gemma*  *Staurophora salina*  *Tryblionella hungarica* | **Cyanobacteria:** |

| Paper 41 – Gao L, Bowker MA, Sun H *et al.* (2020) | |
| --- | --- |
| Country: China Taxonomical Method(s): Not reported | |
| **Algae:** | **Cyanobacteria:**  *Nostoc* spp.  *Phormidium angustissimum* (Currently *Leptolyngbya angustissima*)  *Phormidium tenue* (Currently *Leptolyngbya tenuis*) |
| Paper 42 – Gao L, Sun H, Xu M *et al.* (2020) | |
| Country: China Taxonomical Method(s): Not reported | |
| **Algae:** | **Cyanobacteria:**  *Nostoc* spp.  *Phormidium angustissimum* (Currently *Leptolyngbya angustissima*)  *Phormidium tenue* (Currently *Leptolyngbya tenuis*) |
| Paper 43 – García-Meza JV, Carrillo-Chávez A, Morton-Bermea O (2006) | |
| Country: Mexico Taxonomical Method(s): Morphology | |
| **Algae:**  *Chlorella vulgaris*  *Cholorococcum* sp. | **Cyanobacteria:**  *Anabaena* sp.  *Phormidium* sp.  *Pseudanabaena* sp. |
| Paper 44 – Giraldo-Silva A, Fernandes V, Bethany J *et al.* (2020) | |
| Country: United States (USA) Taxonomical Method(s): Morphology | |
| **Algae:** | **Cyanobacteria:**  *Scytonema* spp.  *Nostoc* spp.  *Tolypothrix* spp. |
| Paper 45 – Giraldo-Silva A, Nelson C, Barger NN *et al.* (2019) | |
| Country: United States (USA) Taxonomical Method(s): Morphology and Molecular Biology | |
| **Algae:**  Bacillariophyta (plastids)  Chlorophyta (plastids) | **Cyanobacteria:**  *Chroococcidiopsis* spp.  *Leptolyngbya* spp.  *Lyngbya* spp.  *Microcoleus steenstrupii*  *Microcoleus vaginatus*  *Nostoc* spp.  *Scytonema* spp.  *Synechococcus* spp.  *Tolypothrix* spp.  *Trichocoleus* spp. |

| Paper 46 – Giraldo-Silva A, Nelson C, Penfold C *et al.* (2020) | |
| --- | --- |
| Country: United States (USA) Taxonomical Method(s): Morphology and Molecular Biology | |
| **Algae:** | **Cyanobacteria:**  *Microcoleus steenstrupii*  *Microcoleus vaginatus*  *Nostoc* spp.  *Scytonema* spp.  *Tolypothrix* spp. |
| Paper 47 – Grishkan I, Kidron GJ. (2013) | |
| Country: Israel Taxonomical Method(s): Not reported | |
| **Algae:** | **Cyanobacteria:**  *Coccobotrys dunense*  *Microcoleus* sp.  *Nostoc* sp.  *Oscillatoria* sp.  *Schizothrix* sp.  *Scytonema* sp. |
| Paper 48 – Gypser S, Herppich WB, Fischer T *et al.* (2016) | |
| Country: Germany Taxonomical Method(s): Morphology | |
| **Algae:**  *Klebsormidium* sp.  *Ulothrix* sp.  *Zygogonium* sp. | **Cyanobacteria:** |

| Paper 49 – Gypser S, Veste M, Fischer T. (2016) | |
| --- | --- |
| Country: Germany Taxonomical Method(s): Morphology | |
| **Algae:**  *Actinochloris sphaerica*  *Acutodesmus obliquus* (Currently *Tetradesmus obliquus*)  *Bracteacoccus* cf. *minor*  *Bracteacoccus* sp.  *Bumilleriopsis* cf. *peterseniana*  *Chlamydomonas* cf. *callunae* (Currently *Heterochlamydomonas* *callunae*)  *Chlamydomonas* cf. *moewusii*  *Chlamydomonas* cf. *reisiglii*  *Chlorella chlorelloides*  *Chlorella vulgaris*  *Chlorococcum* cf. *oleofaciens* (Currently *Pleurastrum insigne*)  *Chloroidium ellipsoideum*  *Chlorokybus atmophyticu*  *Chlorolobion lunulatum*  *Chloromonas actinochloris*  *Chloromonas* cf. *augustae*  *Chloromonas* cf. *reticulata*  *Cylindrocystis crassa*  *Dictyosphaerium* sp.  *Diplosphaera chodatii*  *Elliptochloris subsphaerica*  *Eustigmatos magnus* (Currently *Vischeria magna*)  *Geminella interrupta*  *Graesiella emersonii*  *Interfilum paradoxum*  *Klebsormidium* cf. *nitens*  *Klebsormidium* cf. *subtile*  *Klebsormidium crenulatum*  *Klebsormidium flaccidum*  *Koliella* sp.  *Leptosira* cf. *erumpens*  *Lobochlamys* cf. *culleus*  *Monoraphidium* cf. *pusillum*  *Myrmecia* cf. *biatorellae*  *Pleurochloris meiringensis*  *Podohedra bicaudata*  *Pseudochlorella* sp.  *Scenedesmus* sp.  *Spongiochloris* cf. *incrassata* (Currently *Valeriella* *incrassata*)  *Spongiochloris* cf. *minor* (Currently *Valeriella* *minor*)  *Spongiochloris spongiosa*  *Stichococcus bacillaris*  *Tetracystis sarcinalis*  *Tetracystis* sp.  *Ulothrix* sp.  *Xanthonema* cf. *bristolianum*  *Xanthonema exile*  *Zygogonium* sp. | **Cyanobacteria:** |

| Paper 50 – Hakkoum Z, Minaoui F, Douma M (2020) | |
| --- | --- |
| Country: Morocco Taxonomical Method(s): Morphology | |
| **Algae:** | **Cyanobacteria:**  *Chroococcus* sp.  *Hormoscilla* sp.  *Leptolyngbya foveolarum*  *Leptolyngbya lurida* (Currently *Drouetiella lurida*)  *Leptolyngbya pseudovalderiana*  *Leptolyngbya* sp.  *Leptolyngbya tenuis*  *Lyngbya* sp.  *Nostoc muscorum* (Currently *Desmonostoc muscorum*)  *Phormidium articulatum*  *Phormidium crassivaginatum*  *Phormidium Kuetzingianum*  *Phormidium molle* (Currently *Phormidesmis mollis*)  *Phormidium paulsenianum*  *Phormidium priestley* (Currently *Phormidesmis priestley*)  *Pseudanabaena balatonica*  *Pseudanabaena galeata*  *Pseudanabaena minima*  *Pseudanabaena moniliformis*  *Pseudanabaena* sp.  *Pseudanabaena starmachii*  *Schizothrix* sp.  *Synechocystis* sp. |
| Paper 51 – Hashim ZE, Al-Madhhachi AT, Alzubaidi LA. (2020) | |
| Country: Iraq Taxonomical Method(s): Morphology | |
| **Algae:**  *Oedogonium*  *Spirogyra*  *Zygogonium ericetorum* | **Cyanobacteria:**  *Oscillatoria*  *Phormidium* |

| Paper 52 – Hodac L, Brinkmann N, Mohr K, *et al.* (2015) | |
| --- | --- |
| Country: Germany Taxonomical Method(s): Morphology and Molecular Biology | |
| **Algae:**  *Acutodesmus obliquus* (Currently *Tetradesmus obliquus*)  *Bracteacoccus aerius*  *Bracteacoccus* sp.  *Chlamydomonas* sp.  *Chlamydopodium* sp.  *Chlorella* sp.  *Chlorococcum ellipsoideum*  *Chlorococcum minutum* (Currently *Pleurastrum minutum*)  *Chlorococcum sphacosum* (Currently *Pleurastrum insigne*)  *Coccomyxa* cf. *pringsheimii* (Currently *Pseudococcomyxa pringsheimii*)  *Coccomyxa* cf. *simplex* (Currently *Pseudococcomyxa simplex*)  *Desmochloris* cf. *halophila*  *Desmodesmus* cf. *armatus*  *Dilabifilum printzii* (Currently *Pseudopleurococcus printzii*)  *Elliptochloris subsphaerica*  *Hazenia mirabilis*  *Marvania* sp.  *Monoraphidium* cf.  *Muriella terrestris*  *Mychonastes* cf. *homosphaera*  *Mychonastes* sp.  *Neocystis* cf. *mucosa*  *Pseudendocloniopsis botryoides*  *Pseudendoclonium akinetum* (Currently *Tupiella akineta*)  *Pseudomuriella* cf. *schumacherensis*  *Scenedesmaceae* sp.  *Stichococcus bacillaris*  *Stichococcus* cf. *deasonii* (Currently *Deuterostichococcus marinus*)  *Stichococcus mirabilis*  *Stichococcus* sp.1  *Stichococcus* sp.2  *Stichococcus* sp.3  *Stichococcus* sp.4 | **Cyanobacteria:** |
| Paper 53 – Jia R, Teng J, Chen M *et al.* (2018) | |
| Country: China Taxonomical Method(s): Not reported | |
| **Algae:**  *Navicula cryptocephala* | **Cyanobacteria:** |

| Paper 54 – Karaoz U, Couradeau E, Rocha UN (2018) | |
| --- | --- |
| Country: Israel Taxonomical Method(s): Molecular Biology | |
| **Algae:** | **Cyanobacteria:**  *Calothrix*  *Microcoloeus vaginatus*  *Microcoleus steenstrupii*  *Nostoc*  *Scytonema* |
| Paper 55 – Kheirfam (2020) | |
| Country: Iran Taxonomical Method(s): Morphology | |
| **Algae:** | **Cyanobacteria:**  *Aphanothece*  *Lyngbya*  *Nostoc*  *Oscillatoria*  *Phormidium* |
| Paper 56 – Kheirfam H, Asadzadeh F (2020) | |
| Country: Iran Taxonomical Method(s): Morphology | |
| **Algae:** | **Cyanobacteria:**  *Nostoc* sp.  *Oscillatoria* sp. |
| Paper 57 – Kheirfam H, Roohi M (2020) | |
| Country: Iran Taxonomical Method(s): Morphology and Molecular Biology | |
| **Algae:** | **Cyanobacteria:**  *Nostoc* sp.  *Oscillatoria* sp. |
| Paper 58 – Kidron GJ, Xiao B, Benenson I (2020) | |
| Country: Israel Taxonomical Method(s): Not reported | |
| **Algae:** | **Cyanobacteria:**  *Calothrix* cf. *parietina*  *Microcoleus vaginatus*  *Nostoc* sp.  *Phormidium* sp.  *Scytonema* sp. |
| Paper 59 – Kremer B, Kaźmierczak J, Środoń J (2018) | |
| Country: Ukraine Taxonomical Method(s): Morphology | |
| **Algae:**  *Eremosphaera gigas*  *Nannochloropsis*  *Oocystis viridis*  *Trentepohlia* | **Cyanobacteria:**  *Chroococcidiopsis*  *Entophysalis*  *Myxosarcina*  *Pleurocapsa*  *Xenococcus* |

| Paper 60 – Kuske C, Yeager C, Johnson S *et al.* (2012) | |
| --- | --- |
| Country: United States (USA) Taxonomical Method(s): Molecular Biology | |
| **Algae:** | **Cyanobacteria:**  *Microcoleus vaginatus*  *Nostoc* sp.  *Scytonema* sp.  *Tolypothrix* sp. |
| Paper 61 – Lan S, Thomas AD, Tooth S *et al.* (2021) | |
| Country: Botswana Taxonomical Method(s): Molecular Biology | |
| **Algae:** | **Cyanobacteria:**  *Arthronema africanum*  *Chlorogloea microcystoides*  *Chlorogloea* sp.  *Chroococcidiopsis thermalis*  *Crinalium epipsammum*  *Crinalium* sp.  *Gloeobacter violaceus*  *Leptolyngbya boryana*  *Leptolyngbya foveolarum*  *Lyngbya aestuarii*  *Lyngbya polychroa* (Currently *Lyngbya sordida*)  *Microcoleus paludosus*  *Microcoleus* sp.  *Microcoleus steenstrupii*  *Nostoc* sp.  *Oscillatoria* sp.  *Petalonema* sp.  *Phormidium* sp.  *Pycnacronema brasiliense*  *Pycnacronema savannensis*  *Pycnacronema* sp.  *Scytonema arcangelii*  *Scytonema hyalinum*  *Scytonema ocellatum*  *Scytonema* sp.  *Starria zimbweënsis*  *Stigonema* sp.  *Symploca* sp.  *Symplocastrum torsivum*  *Symplryonema* sp.  *Synechococcus* sp. |
| Paper 62 – Li H, Li R, Rossi F *et al.* (2016) | |
| Country: China Taxonomical Method(s): Molecular Biology | |
| **Algae:** | **Cyanobacteria:**  *Microcoleus* sp. |
| Paper 63 – Li JY, Jin XY, Zhang XC *et al.* (2020) | |
| Country: China Taxonomical Method(s): Molecular Biology | |
| **Algae:** | **Cyanobacteria:**  *Microcoleus*  *Tolypothrix* |

| Paper 64 – Lorite J, Agea D, García-Robles H *et al.* (2019) | |
| --- | --- |
| Country: Spain Taxonomical Method(s): Morphology | |
| **Algae:** | **Cyanobacteria:**  *Aphanocapsa* sp.  *Camptylonemopsis* sp.  *Chlorogloea* sp.  *Microcoleus* sp.  *Nostoc* sp.  *Schizothrix* sp. |
| Paper 65 – Machado-de-Lima NM, Branco LHZ (2020) | |
| Country: Brazil Taxonomical Method(s): Morphology and Molecular Biology | |
| **Algae:** | **Cyanobacteria:**  *Gracilinea arenicola*  *Konicacronema caatingense*  *Marmoreocelis xerophila*  *Pycnacronema caatingensis*  *Pycnacronema edaphicum*  *Trichocoleus caatingensis* |

| Paper 66 – Machado-de-Lima NM, Fernandes VMC, Roush D *et al.* (2019) | |
| --- | --- |
| Country: Brazil Taxonomical Method(s): Molecular Biology | |
| **Algae:** | **Cyanobacteria:**  *Acaryochloris*  *Aetokthonos*  *Brasilonema*  *Calothrix*  *Chakia*  *Chroococcidiopsis*  *Chroococcus*  *Crinalium*  *Demonostoc*  *Fischerella*  *Gloeobacter*  *Gloeocapsa*  *Gloeomargarita*  *Hassalia*  *Hyella*  *Komvophoron*  *Leptolyngbya*  *Mastigocladus*  *Mastigocoleus*  *Microcystis*  *Nostoc*  *Nostochopsis*  *Oculatella*  *Phormidesmis*  *Porphyrosiphon*  *Pleurocapsa*  *Potamolinea*  *Prochlorococcus*  *Pseudanabaena*  *Pseudophormidium*  *Pycnacronema*  *Rivularia*  *Scytonema*  *Stigonema*  *Trichormus* |
| Paper 67 – Machado-de-Lima NM, Muñoz-Rojas M, Vázquez-Campos X *et al.* (2021) | |
| Country: Brazil Taxonomical Method(s): Morphology and Molecular Biology | |
| **Algae:** | **Cyanobacteria:**  *Aetokthonos*  *Brasilonema*  *Chroococcidiopsis*  *Leptolyngbya*  *Microcoleus*  *Microcoleus vaginatus*  *Nostoc*  *Oculatella*  *Porphyrosiphon*  *Potamosiphon*  *Pycnacronema*  *Scytonema*  *Stigonema* |

| Paper 68 – Maier S, Tamm A, Wu D *et al.* (2018) | |
| --- | --- |
| Country: South Africa Taxonomical Method(s): Molecular Biology | |
| **Algae:** | **Cyanobacteria:**  *Chroococcidiopsis*  *Leptolyngbya*  *Microcoleus*  *Nostoc*  *Phormidium*  *Pseudanabaena* |
| Paper 69 – Moreira-Grez B, Tam K, Cross AT *et al.* (2019) | |
| Country: United States (USA) Taxonomical Method(s): Molecular Biology | |
| **Algae:** | **Cyanobacteria:**  *Acaryochloris marina*  *Arthrospira platensis*  *Chamaesiphon* sp.  *Chlorogloeopsis fritschii*  *Chroococcidiopsis thermalis*  *Crinalium epipsammum*  *Crocosphaera watsonii* (Currently *Cyanobium waterburyi*)  *Cyanobacterium* sp.  *Cyanobacterium stanieri*  *Cyanobium gracile*  *Cyanothece* sp.  *Dactylococcopsis salina*  *Fischerella muscicola*  *Geitlerinema* sp.  *Gloeocapsa* sp.  *Gloeobacter violaceus*  *Halothece* sp.  *Leptolyngbya* sp.  *Lyngbya majuscula*  *Lyngbya* sp.  *Microcoleus chthonoplastes* (currently *Coleofasciculus chthonoplastes*)  *Microcoleus* sp.  *Myxosarcina* sp.  *Oscillatoria* sp.  *Pleurocapsa* sp.  *Prochlorococcus* sp.  *Prochloron didemni*  *Prochlorothrix hollandica*  *Rivularia* sp.  *Rubidibacter lacunae*  *Spirulina* sp.  *Stanieria cyanosphaera*  *Synechococcus* sp.  *Synechocystis* sp.  *Thermosynechococcus elongatus* (Currently *Thermosynechococcus vestitus*)  *Trichodesmium erythraeum*  *Xenococcus* sp. |

| Paper 70 – Moya P, Molins A, Chiva S, *et al*. (2020) | |
| --- | --- |
| Country: Spain Taxonomical Method(s): Molecular Biology | |
| **Algae:**  *Trebouxia asymmetrica*  *Trebouxia* sp. OTU A25  *Trebouxia cretacea*  *Trebouxia* sp. OTU I53  *Trebouxia vagua*  *Myrmecia israeliensis*  *Bracteacoccus* sp.  *Vulcanochloris* sp. | **Cyanobacteria:** |
| Paper 71 – Mugnai G, Rossi F, Chamizo S *et al.* (2020) | |
| Country: Israel Taxonomical Method(s): Not reported | |
| **Algae:** | **Cyanobacteria:**  *Leptolyngbya ohadii* |
| Paper 72 – Mugnai G, Rossi F, Felde VJMNL *et al.* (2018) | |
| Country: China Taxonomical Method(s): Morphology and Molecular Biology | |
| **Algae:** | **Cyanobacteria:**  *Schizothrix* cf. *delicatissima* |
| Paper 73 – Muñoz-Martín MÁ, Becerra-Absalón I, Perona E *et al.* (2019) | |
| Country: Spain Taxonomical Method(s): Morphology and Molecular Biology | |
| **Algae:** | **Cyanobacteria:**  *Chroococcidiopsis*  *Kastovskya*  *Leptolyngbya frigida* (currently *Stenomitos frigidus*)  *Leptolyngbya* sp.  *Microcoleus sociatus*  *Microcoleus* sp.  *Microcoleus steenstrupii*  *Microcoleus vaginatus*  *Oculatella*  *Phormidium*  *Phormidium ambiguum*  *Phormidium murrayi* (Currently *Wilmottia murrayi*)  *Schizothrix*  *Schizothrix.* cf. *calcicola*  *Tolypothrix* sp.  *Trichocoleus*  *Trichocoleus desertorum* |
| Paper 74 – Muñoz-Rojas M, Román JR, Roncero-Ramos B *et al.* (2018) | |
| Country: Australia Taxonomical Method(s): Not reported | |
| **Algae:** | **Cyanobacteria:**  *Nostoc commune*  *Scytonema hyalinum*  *Tolypothrix distorta* |

| Paper 75 – Muñoz-Rojas M, Chilton A, Liyanage G *et al.* (2018) | |
| --- | --- |
| Country: Australia Taxonomical Method(s): Morphology and Molecular Biology | |
| **Algae:** | **Cyanobacteria:**  *Acaryochloris* sp.  *Anabaena* spp.  *Aphanizomenon ovalisporum* (Currently *Umezakia ovalisporum*)  *Arthronema* sp.  *Brasilonema* spp.  *Brasilonema terrestre*  *Calothrix brevissima*  *Calothrix desertica* (Currently *Dulcicalothrix desertica*)  *Calothrix* sp.  *Chamaesiphon minutus*  *Chamaesiphon subglobosus Cylindrospermum stagnale*  *Leptolyngbya antarctica* (Currently *Shackletoniella antarctica*)  *Leptolyngbya foveolarum*  *Leptolyngbya* sp.  *Limnothrix* sp.  *Microcoleus paludosus*  *Nostoc microscopicum*  *Nostoc muscorum* (Currently *Desmonostoc muscorum*)  *Nostoc* spp.  *Oscillatoria acuminata* (Currently *Oxynema acuminatum*)  *Oscillatoria rosea* (Currently *Phormidium roseum* or *Limnothrix rosea*)  *Phormidium murrayi* (Currently *Wilmottia murrayi*)  *Phormidium pseudopriestleyi*  *Phormidium* sp.  *Scytonema* sp.  *Stanieria cyanosphaera*  *Synechococcus elongatus*  *Tolypothrix distorta* |

| Paper 76 – Nelson C, Giraldo-Silva A, Garcia-Pichel F (2020) | |
| --- | --- |
| Country: United States (USA) Taxonomical Method(s): Molecular Biology | |
| **Algae:** | **Cyanobacteria:**  *Anabaena* sp.  *Aphanothece* sp.  *Crinalium* sp.  *Leptolyngbya* sp.  *Lyngbya* sp.  *Microcoleus paludosus*  *Microcoleus steentrupii*  *Microcoleus vaginatus*  *Nostoc* sp.  *Oscillatoria* sp.  *Porphyrosiphon* sp.  *Potamolinea* sp.  *Pseudanabaena* sp.  *Pycnacronema* sp.  *Schizothrix* sp.  *Scytonema* sp.  *Tolypothrix* sp. |
| Paper 77 – Nejidat A, Potrafka RM, Zaady E (2016) | |
| Country: Israel Taxonomical Method(s): Molecular Biology | |
| **Algae:** | **Cyanobacteria:**  *Microcoleus sociatus*  *Microcoleus steenstrupii*  *Microcoleus vaginatus*  *Nostoc* spp.  *Schizothrix*  *Scytonema* spp.  *Tolypothrix* |

| Paper 78 –Nowicka-Krawczyk P, Żelazna-Wieczorek J, Otlewska A, *et al.* (2014) | |
| --- | --- |
| Country: Poland Taxonomical Method(s): Morphology | |
| **Algae:**  *Achnanthidium minutissimum*  *Anomoeoneis vitrea* (Currently *Brachysira vitrea*)  *Apatococcus lobatus*  *Chlorella* sp*.*  *Chlorella vulgaris*  *Chlorococcum infusionum*  *Cocconeis placentula* var. *lineata* (Currently *Cocconeis lineata*)  *Cocconeis pseudothumensis*  *Coenochloris* sp.  *Denticula kuetzingii*  *Diadesmis contenta* (Currently *Humidophila contenta*)  *Epithemia turgida* var. *granulata*  *Euastrum* sp.  *Gomphonema parvulum*  *Luticola mutica*  *Navicula cryptocephala*  *Neidium ampliatum*  *Nitzschia debilis* (Currently *Tryblionella debilis*)  *Nitzschia fonticola*  *Nitzschia palea*  *Nitzschia vitrea*  *Phacotus* sp.  *Pinnularia borealis*  *Planothidium lanceolatum*  *Trebouxia* sp.  *Trentepohlia* cf. *odorata*  *Trybionella hungarica*  *Xanthonema montanum*  *Xanthonema* sp*.* | **Cyanobacteria:**  *Chroococcopsis* cf. *fluviatilis*  *Chroococcus minor*  *Chroococcus* sp.  *Chroococcus varius*  *Cyanobium* cf. *parvum*  *Gloeocapsa* sp.  *Gloeothece palea*  *Gloeothece* sp.  *Leptolyngbya foveolarum*  *Leptolyngbya notata*  *Leptolyngbya frigida* (Currently *Stenomitos frigidus*)  *Microcoleus vaginatus*  *Nostoc commune*  *Nostoc microscopicum*  *Nostoc punctiforme*  *Nostoc* sp.  *Oscillatoria* cf. *princeps*  *Phormidium aerugineocaeruleum* (Currently *Potamolinea aerugineocaerulea*)  *Phormidium breve*  *Phormidium tergestinum*  *Scytonema drilosiphon*  *Tolypothrix* sp. |
| Paper 79 – Ochoa-Hueso R, Mondragon-Cortés T, Concostrina-Zubiri L *et al.* (2017) | |
| Country: Spain Taxonomical Method(s): Morphology | |
| **Algae:** | **Cyanobacteria:**  *Calothrix*  *Leptolyngbya*  *Microcoleus*  *Nostoc*  *Phormidium*  *Scytonema* |

| Paper 80 – Ouyang H, Hu C (2017) | |
| --- | --- |
| Country: China Taxonomical Method(s): Not reported | |
| **Algae:** | **Cyanobacteria:**  *Nostoc*  *Microcoleus*  *Scytonema* |
| Paper 81 – Panigada C, Tagliabue G, Zaady E *et al.* (2019) | |
| Country: Israel Taxonomical Method(s): Not reported | |
| **Algae:** | **Cyanobacteria:**  *Calothrix* spp.  *Chroococcus* sp.  *Microcoleus vaginatus*  *Nostoc punctiforme* |
| Paper 82 – Pombubpa N, Kurbessoian T, Stajich JE *et al.* (2020) | |
| Country: United States (USA) Taxonomical Method(s): Molecular Biology | |
| **Algae:**  cf. *Actinochloris* sp.  *Bracteacoccus* sp.  *Chlorosarcinopsis* sp.  *Myrmecia* sp.  *Stichococcus* sp.  *Parietochloris* sp. | **Cyanobacteria:**  *Hassallia* sp.  *Mojavia pulchra*  *Nostoc* sp.  *Oculatella coburnii*  *Roholtiella mohavensis*  *Spirirestis rafaelensis*  *Symplocastrum flechtnerae*  *Trichocoleus desertorum* |
| Paper 83 – Pombubpa N, Pietrasiak N, Ley PD *et al.* (2020) | |
| Country: United States (USA) Taxonomical Method(s): Molecular Biology | |
| **Algae:** | **Cyanobacteria:**  *Calothrix* sp  *Chroococcidiopsis* sp  *Microcoleus* sp |

| Paper 84 – Pushkareva E, Baumann K, Van AT *et al.* (2021) | |
| --- | --- |
| Country: Iceland Taxonomical Method(s): Morphology | |
| **Algae:**  *Achnanthes*  *Achnanthidium*  *Actinella*  *Amphora*  *Asterionella*  *Aulacoseira*  *Brachysira*  *Bracteacoccus* cf. *giganteus*  *Bracteacoccus* sp.  *Caloneis*  *Cavinula*  *Chlamydomonas* cf. *asymmetrica Chlamydomonas* sp.  *Chlorella vulgaris*  *Chlorococcum* sp.  *Chlorolobion* sp.  *Coccomyxa* sp.  *Cocconeis*  *Coelastrella* sp.  *Coelastrella terrestris Heterochlamydomonas* cf. *callunae Neocystis* sp.  *Cosmarium* cf. *anceps*  *Cosmarium* sp.  *Cyclotella*  *Cymbella*  *Cymbopleura*  *Desmococcus* cf. *olivaceus*  *Desmogonium*  *Diadesmis*  *Diatoma*  *Diploneis*  *Diplosphaera* sp.  *Elliptochloris subsphaerica*  *Encyonema*  *Encyonopsis*  *Eolimna*  *Epithemia*  *Eucocconeis*  *Eunotia*  *Fallacia*  *Fragilaria*  *Fragilariforma*  *Frickea*  *Frustulia*  *Geissleria*  *Gloeocystis* cf. *polydermatica* (Currently *Sporotetras polydermatica*)  *Gomphonema*  *Halamphora*  *Hantzschia*  *Heterococcus* sp.  *Hippodonta*  *Karayevia*  *Klebsormidium* cf. *delicatum* | **Cyanobacteria:**  *Microcoleus vaginatus* |

Pushkareva E, Baumann K, Van AT *et al.* (2021)…Continuation

| **Algae:**  *Klebsormidium* cf. *flaccidum*  *Klebsormidium* cf. *subtile*  *Klebsormidium* cf. *nitens*  *Kurtkrammeria*  *Lacustriella*  *Leptosira erumpens*  *Luticola*  *Mayamaea*  *Melosira*  *Meridion*  *Muelleria*  *Myrmecia* cf. *bisecta*  *Myrmecia* sp.  *Nannochloris* sp.  *Navicula*  *Neidium*  *Nitzschia*  *Odontidium*  *Parietochloris* cf. *bilobata*  *Pinnularia*  *Placoneis*  *Planothidium*  *Platessa*  *Pleurochloris* sp.  *Psammothidium*  *Pseudomuriella* sp.  *Pseudostaurosira*  *Reimeria*  *Rhopalodia*  *Sellaphora*  *Seminavis*  *Stauroforma*  *Stauroneis*  *Staurosira*  *Staurosirella*  *Stephanodiscus*  *Stichococcus* cf. *bacillaris*  *Stichococcus* cf. *minutus*  *Surirella*  *Tabellaria*  *Tabularia*  *Tetracystis* cf. *sarcinalis*  *Tetracystis* sp.  *Ulnaria*  *Vischeria* cf. *vischeri*  *Xanthonema* cf. *exile*  *Xanthonema* cf. *monochloron*  *Xerochlorella minuta* | **Cyanobacteria:** |
| --- | --- |
| Paper 85 – Rippin M, Borchhardt N, Karsten U *et al.* (2019) | |
| Country: Antarctic and Arctic Taxonomical Method(s): Molecular Biology | |
| **Algae:**  *Klebsormidium dissectum*  *Klebsormidium flaccidum* | **Cyanobacteria:** |

| Paper 86 –Rippin M, Lange S, Sausen N, *et al.* (2018) | |
| --- | --- |
| Country: Norway and Iceland Taxonomical Method(s): Morphology and Molecular Biology | |
| **Algae:**  *Amphora*  *Botrydiopsis*  *Bracteacoccus*  *Cosmarium*  *Chlamydomonas*  *Chlamydopodium*  *Chlorococcum*  *Chloroidium*  *Chloromonas*  *Chromochloris*  *Chrysamoeba*  *Coccomyxa*  *Cylindrocystis*  *Desmococcus*  *Dictyochloropsis*  *Diplosphaera*  *Elliptochloris*  *Eustigmatos*  *Gloeodinium*  *Hantzschia*  *Heterococcus*  *Klebsormidium*  *Leptosira*  *Lobosphaeropsis*  *Monodus*  *Myrmecia*  *Navicula*  *Neidium*  *Neocystis*  *Nitzschia*  *Ochromonas*  *Pinnularia*  *Planophila*  *Pleurochloris*  *Prasiola*  *Pseudochlorella*  *Pseudococcomyxa*  *Rosenvingiella*  *Spumella*  *Stauroneis*  *Stichococcus*  *Trebouxia*  *Trentepohlia*  *Watanabea*  *Xylochloris* | **Cyanobacteria:**  *Aphanothece*  *Coleodesmium*  *Gloeobacter*  *Leptolyngbya*  *Microcoleus*  *Nostoc*  *Oscillatoria*  *Phormidium*  *Pseudanabaena* |

| Paper 87 – Rocha F, Lucas-Borja ME, Pereira P *et al.* (2020) | |
| --- | --- |
| Country: Portugal Taxonomical Method(s): Not reported | |
| **Algae:** | **Cyanobacteria:**  *Anabaena* sp.  *Nostoc* sp. |
| Paper 88 – Román JR, Chilton AM, Cantón Y *et al.* (2020) | |
| Country: Australia Taxonomical Method(s): Not reported | |
| **Algae:** | **Cyanobacteria:**  *Leptolyngbya* sp.  *Nostoc* sp.  *Scytonema* sp. |
| Paper 89 – Román JR, Roncero-Ramos B, Chamizo S *et al.* (2018) | |
| Country: Spain Taxonomical Method(s): Not reported | |
| **Algae:** | **Cyanobacteria:**  *Nostoc commune*  *Scytonema hyalinum*  *Tolypothrix distorta* |

| Paper 90 – Romero ALN, Moratta MAH, Vento B *et al.* (2019) | |
| --- | --- |
| Country: Argentina Taxonomical Method(s): Morphology | |
| **Algae:** | **Cyanobacteria:**  *Anabaena anomala* (Currently *Trichormus anomalus*)  *Anabaena circinalis* (Currently *Dolichospermum circinale*)  *Cylindrospermum* sp.  *Lyngbya borgerti*  *Lyngbya cryptovaginata* (Currently *Limnoraphis cryptovaginata*)  *Lyngbya* sp.  *Microcoleus vaginatus*  *Nodularia* sp.  *Nostoc calcicola*  *Nostoc commune*  *Nostoc entophytum* (Currently *Nostoc paludosum*)  *Nostoc muscorum* (Currently *Desmonostoc muscorum*)  *Nostoc punctiforme*  *Nostoc* sp.  *Oscillatoria obscura*  *Oscillatoria pseudogeminata* (Currently *Jaaginema pseudogeminatum*)  *Oscillatoria* sp.  *Oscillatoria subbrevis*  *Phormidium angustissimum* (Currently *Leptolyngbya angustissima*)  *Phormidium bohneri*  *Phormidium fragile*  *Phormidium molle* (Currently *Phormidesmis mollis*)  *Phormidium rubroterricola*  *Schizothrix beccarii*  *Scytonema coactile*  *Scytonema millei*  *Scytonema schmidtii*  *Scytonema tolypothrichoides*  *Tolypothrix phyllophila* |

| Paper 91 – Roncero-Ramos B, Muñoz-Martín MA, Cantón Y *et al.* (2020) | |
| --- | --- |
| Country: Spain Taxonomical Method(s): Morphology and Molecular Biology | |
| **Algae:** | **Cyanobacteria:**  *Chroococcidiopsis* sp.  *Leptolyngbya frigida* (currently *Stenomitos frigidus*)  *Leptolyngbya* sp.  *Macrochaete lichenoides*  *Microcoleus paludosus*  *Microcoleus* sp.  *Microcoleus steenstrupii*  *Microcoleus vaginatus*  *Mojavia* sp.  *Nostoc commune*  *Oculatella kazantipica*  *Oculatella* sp.  *Phormidium* sp.  *Pleurocapsa* sp.  *Scytonema hyalinum*  *Scytonema* sp.  *Tolypothrix distorta*  *Trichocoleus desertorum* |
| Paper 92 – Roncero-Ramos B, Muñoz-Martín MÁ, Chamizo S *et al.* (2019) | |
| Country: Spain Taxonomical Method(s): Morphology and Molecular Biology | |
| **Algae:** | **Cyanobacteria:**  *Leptolyngbya frigida* (currently *Stenomitos frigidus*)  *Microcoleus steenstrupii*  *Nostoc calcicola*  *Nostoc commune*  *Oculatella kazantipica*  *Schizothrix* cf. *calcicola*  *Scytonema hyalinum*  *Tolypothrix distorta*  *Trichocoleus desertorum* |

| Paper 93 – Samolov E, Baumann K, Büdel B *et al.* (2020) | |
| --- | --- |
| Country: Chile Taxonomical Method(s): Morphology and Molecular Biology | |
| **Algae:**  *Botrydiopsis* cf. *constricta*  *Botrydiopsis* cf. *intercedens*  *Bracteacoccus bullatus*  *Bracteacoccus medionucleatus*  *Bracteacoccus* sp.  *Chlamydomonas* cf. *pseudoelegans Chlamydomonas* sp.  *Chlorella* sp.  *Chlorococcum* cf. *minimum*  *Chlorococcum* cf. *oleofaciens* (Currently *Pleurastrum insigne*)  *Chlorococcum echinozygotum* (Currently *Chlorococcum infusionum*)  *Chloroidium* sp.  *Chlorosarcinopsis* cf. *gelatinosa*  *Fasciculochloris* sp.  *Heterochlamydomonas* cf. *inaequalis*  *Heterochlamydomonas* sp.  *Coccomyxa simplex* (Currently *Pseudococcomyxa simplex*)  *Cylindrocystis brebissonii*  *Cylindrocystis crassa*  *Desmococcus* sp.  *Diplosphaera chodatii*  *Edaphochlorella mirabilis*  *Elliptochloris perforata*  *Elliptochloris subsphaerica*  *Gloeocystis* cf. *vesiculosa*  *Gloeocystis* sp.  *Interfilum massjukiae*  *Interfilum* sp.  *Ixipapillifera* sp.  *Keratococcus raphidioides*  *Klebsormidium delicatum var. americanum*  *Klebsormidium delicatum var. deserticum*  *Klebsormidium deserticola*  *Klebsormidium fluitans*  *Klebsormidium nitens*  *Klebsormidium* sp.  *Klebsormidium sylvaticum*  *Leptosira* cf. *erumpens*  *Lobochlamys segnis*  *Lobosphaera incisa*  *Macrochloris* sp.  *Myrmecia* cf. *astigmatica*  *Myrmecia* cf. *bisecta*  *Neocystis* cf. *brevis*  *Neospongiococcum* cf. *excentricum Pseudomuriella aurantiaca*  *Parietochloris* cf. *pseudoalveolaris*  *Stichococcus* sp.  *Tetracystis intermedia*  *Trebouxia* sp.  *Vischeria magna*  *Vischeria vischeri*  *Watanabea borysthenica*  *Xanthonema exile*  *Xerochlorella minuta* | **Cyanobacteria:**  *Chroococcidiopsis* sp.  *Leptolyngbya henningsii* (Currently *Phormidium henningsii*)  *Leptolyngbya* sp.  *Leptolyngbya tenuis*  *Microcoleus* sp.  *Microcoleus vaginatus*  *Myxacorys* sp.  *Nodosilinea epilithica*  *Nostoc* cf. *edaphicum*  *Nostoc* cf. *punctiforme*  *Nostoc* sp.  *Oscillatoria* cf. *tenuis*  *Phormidesmis* sp.  *Phormidium* sp.  *Pleurocapsa minor*  *Pseudophormidium* cf. *hollerbachianum Stenomitos* sp.  *Trichocoleus* cf. *badius*  *Trichocoleus sociatus* (Currently *Funiculus sociatus*) |

| Paper 94 – Schulz K, Mikhailyuk T, Dreßler M *et al.* (2016) | |
| --- | --- |
| Country: Germany Taxonomical Method(s): Morphology | |
| **Algae:**  *Achnanthes coarctata*  *Actinochloris sphaerica*  *Actinocyclus* sp.  *Acutodesmus obliquus* (Currently *Tetradesmus obliquus*)  *Amphora* cf. *indistincta*  *Amphora indistinct*  *Bracteacoccus* cf. *minor*  *Bracteacoccus* sp.  *Bumilleriopsis* cf. *peterseniana*  *Caloneis amphisbaena*  *Carteria* cf. *crucifera* (Currently *Pseudagloë crucifera*)  *Catenula adhaerens*  *Chlamydomonas* cf. *callunae* (Currently *Heterochlamydomonas* *callunae*) *Chlamydomonas* cf. *moewusii Chlamydomonas* cf. *reisiglii*  *Chlorella chlorelloides*  *Chlorella vulgaris*  *Chlorococcum* cf. *oleofaciens* (Currently *Pleurastrum insigne*)  *Chloroidium ellipsoideum*  *Chlorokybus atmophyticus*  *Chlorolobion lunulatum*  *Chloromonas actinochloris*  *Chloromonas* cf. *augustae*  *Chloromonas* cf. *reticulata*  *Cocconeis* cf. *neothumensis*  *Cocconeis* cf. *neothumensis*  *Cocconeis neothumensis*  *Cocconeis placentula*  *Cocconeis placentula var. euglypta* (Currently *Cocconeis euglypta*)  *Cocconeis scutellum var. scutellum Planothidium delicatulum*  *Cylindrocystis crassa*  *Dictyosphaerium* sp.  *Diplosphaera chodatii*  *Elliptochloris subsphaerica*  *Epithemia* cf. *turgida*  *Eustigmatos magnus* (Currently *Vischeria magna*)  *Fallacia* cf. *floriniae*  *Fallacia clepsidroides*  *Fallacia tenera* (Currently *Pseudofallacia tenera*)  *Fistulifera* cf. *pelliculosa*  *Fragilaria martyi* (Currently *Staurosirella martyi*)  *Fragilaria schulzii* (Currently *Martyana schulzii*)  *Geminella interrupta*  *Graesiella emersonii*  *Hantzschia abundans* | **Cyanobacteria:**  *Calothrix* cf. *elenkinii*  *Chroococcus helveticus*  *Coleofasciculus* sp.  *Hassallia* sp.  *Hydrocoryne* sp.  *Leptolyngbya* cf. *henningsii* (Currently *Phormidium henningsii*)  *Leptolyngbya* cf. *notata*  *Leptolyngbya edaphica* (Currently *Timaviella edaphica*)  *Lyngbya* sp.  *Microcoleus vaginatus*  *Nodosilinea* sp.  *Nostoc* cf. *commune*  *Nostoc* cf. *edaphicum*  *Nostoc* cf. *linckia*  *Nostoc* sp.  *Phormidium* cf. *corium*  *Pseudophormidium hollerbachianum Tolypothrix* cf. *tenuis*  *Trichocoleus* sp. |

Schulz K, Mikhailyuk T, Dreßler M *et al.* (2016)… Continuation

| **Algae:**  *Hantzschia amphioxys*  *Hantzschia* sp.  *Interfilum paradoxum*  *Klebsormidium* cf. *nitens*  *Klebsormidium* cf. *subtile*  *Klebsormidium crenulatum*  *Klebsormidium flaccidum*  *Koliella* sp.  *Leptosira* cf. *erumpens*  *Lobochlamys* cf. *culleus*  *Lobochlamys* sp.  *Luticola cohnii*  *Luticola* sp.  *Mayamaea atomus* var*. atomus* (Currently *Mayamaea atomus*)  *Monoraphidium* cf. *pusillum*  *Muelleria* sp.  *Myrmecia* cf. *biatorellae*  *Navicula* cf. *paul-schulzii*  *Navicula* cf. *syvertsenii*  *Navicula viminoides*  *Neochloris* cf. *gelatinosa*  *Opephora burchardtiae* (Currently *Neofragilaria burchardtiae*)  *Opephora* cf. *minuta* (Currently *Pseudostaurosira minuta*)  *Opephora* sp.  *Pinnularia* aff*. intermedia*  *Pinnularia borealis*  *Pinnularia intermedia*  *Placoneis clementis*  *Planothidium lemmermannii* (Currently *Achnanthes lemmermannii*)  *Planothidium* sp.  *Pleurochloris meiringensis*  *Podohedra bicaudata*  *Pseudochlorella* sp.  *Pseudococcomyxa* cf. *simplex*  *Scenedesmus* sp.  *Spongiochloris* cf. *incrassata* (Currently *Valeriella* *incrassata*)  *Spongiochloris* cf. *minor* (Currently *Valeriella* *minor*)  *Spongiochloris spongiosa*  *Staurophora* sp.  *Stichococcus bacillaris*  *Tetracystis sarcinalis*  *Tetracystis* sp.  *Vischeria helvetica*  *Xanthonema* cf. *bristolianum*  *Xanthonema exile* | **Cyanobacteria:** |
| --- | --- |

| Paper 95 – Sepehr A, Hasssanzadeh M, Rodriguez-Caballero E (2019) | |
| --- | --- |
| Country: Iran Taxonomical Method(s): Morphology | |
| **Algae:** | **Cyanobacteria:**  *Anabaena* sp.  *Asterocapsa* cf. *belizensis*  *Gloeocapsa magma* (Currently *Gloeocapsopsis magma*)  *Leptolyngbya boryana*  *Leptolyngbya* cf. *tenerrima*  *Leptolyngbya* sp.  *Microcoleus vaginatus*  *Nostoc commune*  *Nostoc desertorum*  *Nostoc indistinguendum*  *Nostoc membranaceum*  *Oscillatoria annae*  *Oscillatoria irrigua* (Currently *Phormidium irriguum*)  *Oscillatoria splendida* (Currently *Geitlerinema splendidum*)  *Oscillatoria tenuis*  *Phormidium chalybeum*  *Phormidium favosum* (Currently *Microcoleus favosus*)  *Phormidium tergestinum*  *Phormidium uncinatum*  *Pseudanabaena* sp.  *Tolypothrix* sp. |

| Paper 96 – Sommer V, Karsten U, Glaser K. (2020) | |
| --- | --- |
| Country: Germany Taxonomical Method(s): Morphology | |
| **Algae:**  *Apatococcus lobatus*  *Borodinellopsis texensis*  *Bracteacoccus minor*  *Chlorella* sp.  *Chloridella neglecta*  *Chlorococcum* sp.  *Chloroidium ellipsoideum*  *Chlorosarcinopsis minuta* (Currently *Neochlorosarcina minuta*)  *Coelastrella-*like  *Cylindrocystis* sp.  Cymbellaceae  *Diplosphaera chodatii*  *Elliptochloris subsphaerica*  *Gloeobotrys* sp.  *Gloeocystis-*like  *Hantzschia* sp.  *Luticola* sp.  *Mesotaenium* cf. *chlamydosporum*  *Myrmecia bisecta*  *Nannochloris* sp.  *Navicula* sp.  *Parietochloris cohaerens*  *Planophila laetevirens*  *Pseudendoclonium* sp.  *Pleurastrum terricola*  *Spongiochloris excentrica* (Currently *Valeriella excentrica*)  *Stichococcus bacillaris*  *Stichococcus* cf. *allas* (Currently *Deuterostichococcus tetrallantoideus*)  *Stichococcus exiguus* (Currently *Pseudostichococcus monallantoides var. exiguus*)  *Surirella* sp.  *Tetracystis* sp.  *Ulothrix aequalis* | **Cyanobacteria:**  *Gloeocapsa* sp.  *Leptolyngbya* cf. *boryana*  *Leptolyngbya* sp.  *Leptolyngbya-like*  *Microcoleus vaginatus*  *Microcoleus-*like  *Nostoc* sp.  *Oculatella* sp.  *Pseudophormidium edaphicum* (Currently *Timaviella edaphica*)  *Pseudophormidium edaphicum-*like (Currently *Timaviella edaphica*) |

| Paper 97 – Sommer V, Mikhailyuk T, Glaser K *et al.* (2020) | |
| --- | --- |
| Country: Germany Taxonomical Method(s): Morphology and Molecular Biology | |
| **Algae:**  *Alvikia* sp.  *Axilosphaera*  *Borodinellopsis* sp.  *Borodinellopsis texensis*  *Bracteacoccus minor*  *Chlorella* cf. *pituita*  *Chlorogonium*  *Chloroidium saccharophilum*  *Chloroidium* sp.  *Chloromonas* sp.  *Desmochloris* cf. *halophila Halochlorococcum* sp.  *Diplosphaera chodatii*  *Nannochloris* sp.  *Planophila* sp.  *Pseudochlorella signiensis Pseudostichococcus monallantoides Watanabea* sp.  *Spongiococcum*  *Tetradesmus dissociatus* | **Cyanobacteria:**  *Cyanocohniella* sp.  *Leptolyngbya* sp.  *Nodosilinea bijugata*  *Nodosilinea* cf. *signiensis*  *Nodosilinea* sp.  *Nostoc* sp.  *Phormidesmis* sp.  *Pseudophormidium battersii*  *Pseudophormidium edaphicum* (Currently *Timaviella edaphica*) |
| Paper 98 – Sorochkina K, Ayuso SV, Garcia-Pichel F (2018) | |
| Country: United States (USA) Taxonomical Method(s): Morphology | |
| **Algae:**  *Klebsormidium* sp. | **Cyanobacteria:**  *Leptolyngbya* spp.  *Microcoleus steenstrupii*  *Microcoleus vaginatus*  *Scytonema* spp.  *Schizothrix* spp. |
| Paper 99 – Steven B, Belnap J, Kuske CR. (2018) | |
| Country: United States (USA) Taxonomical Method(s): Molecular Biology | |
| **Algae:** | **Cyanobacteria:**  *Microcoleus vaginatus*  *Microcoleus* sp. |
| Paper 100 – Steven B, Gallegos-Graves LV, Yeager CM *et al.* (2012) | |
| Country: United States (USA) Taxonomical Method(s):Molecular Biology | |
| **Algae:** | **Cyanobacteria:**  *Acaryochloris marina*  *Anabaena variabilis* (Currently *Trichormus variabilis*)  *Arthrospira platensis*  *Calothrix desertica* (Currently *Dulcicalothrix desertica*)  *Cyanothece* sp.  *Leptolyngbya* sp.  *Microcoleus chthonoplastes* (currently *Coleofasciculus chthonoplastes*)  *Microcoleus vaginatus*  *Nostoc* sp.  *Phormidium* sp.  *Spirulina* sp.  *Synechococcus* sp.  *Tolypothrix* sp. |

| Paper 101 – Steven B, Gallegos-Graves LV, Belnap J *et al.* (2013) | |
| --- | --- |
| Country: United States (USA) Taxonomical Method(s): Molecular Biology | |
| **Algae:** | **Cyanobacteria:**  *Microcoleus vaginatus*  *Scytonema hyalinum* |
| Paper 102 – Steven B, Yeager C, Belnap J *et al.* (2014) | |
| Country: United States (USA) Taxonomical Method(s): Molecular Biology | |
| **Algae:** | **Cyanobacteria:**  *Microcoleus vaginatus*  *Tolypothrix* sp.  *Trichocoleus sociatus* (Currently *Funiculus sociatus*) |
| Paper 103 – Stricker E, Crain G, Rudgers J, *et al*. (2021) | |
| Country: United States (USA) Taxonomical Method(s): Molecular Biology | |
| **Algae:** | **Cyanobacteria:**  *Microcoleus vaginatus;*  *Microcoleus steenstrupii*  *Microcoleus paludosus*  *Microcoleus chthonoplastes* (Currently *Coleofasciculus chthonoplastes*)  *Leptolyngbya*  *Lyngbya*  *Chroococcidiopsis*  *Crinalium*  *Scytonema*  *Nostoc*  *Calothrix*  *Cephalothrix*  *Tolypothrix* |
| Paper 104 – Szyja M, Menezes AGS, Oliveira FDA *et al.* (2019) | |
| Country: Brazil Taxonomical Method(s): Morphology | |
| **Algae:**  *Chlorella* sp.  *Cylindrocystis brebissoni*  *Desmococcus* sp.  *Follicularia* sp.  *Heterococcus* sp.  *Klebsormidium* sp.  *Neochloris* sp.  *Scenedesmus* sp.  *Scotiellopsis* cf. *rubescens* (Currently *Coelastrella rubescens*)  *Spongiochloris* sp.  *Stichococcus* sp.  Trebouxiophyceae | **Cyanobacteria:**  *Aphanocapsa* sp.  *Calothrix* sp.  *Chroococcidiopsis* sp.  *Gloeocapsa* sp.  *Leptolyngbya* sp.  *Macrochaete lichenoides*  *Microcoleus vaginatus*  *Nostoc edaphicum*  *Nostoc ellipsoideum*  *Nostoc* sp.  *Oscillatoria* sp.  *Pseudophormidium* sp.  *Schizothrix* sp.  *Scytonema hyalinum*  *Tolypothrix* sp. |

| Paper 105 – Tamm A, Caesar J, Kunz N *et al.* (2018) | |
| --- | --- |
| Country: South Africa Taxonomical Method(s): Not reported | |
| **Algae:** | **Cyanobacteria:**  *Chroococcidiopsis*  *Leptolyngbya*  *Microcoleus*  *Nostoc*  *Phormidium*  *Pseudanabaena* |
| Paper 106 – Vinoth M, Sivasankari S, Ahamed A *et al.* (2020) | |
| Country: India Taxonomical Method(s): Morphology | |
| **Algae:** | **Cyanobacteria:**  *Anabaena*  *Anabaena khannae* (Currently *Trichormus khannae*)  *Aphanocapsa*  *Arthrospira*  *Aulosira*  *Chroococcus*  *Gloeocapsa*  *Haplosiphon* (Currently *Scytosiphon*)  *Hydrocoleum*  *Lyngbya cryptovaginata* (Currently *Limnoraphis cryptovaginata*)  *Lyngbya* sp.  *Microchaete*  *Microcoleus*  *Microcystis*  *Nostoc calcicola*  *Nostoc* sp.  *Oscillatoria*  *Oscillatoria formosa* (Currently *Kamptonema formosum*)  *Oscillatoria jasorvensis* (Currently *Kamptonema jasorvense*)  *Oscillatoria limosa*  *Oscillatoria subbrevis*  *Phormidium*  *Plectonema*  *Schizothrix*  *Scytonema*  *Scytonema hofmanni*  *Synechococcus*  *Tolyporthrix* |

| Paper 107 – Wang L, Zhang G, Zhu L *et al.* (2017) | |
| --- | --- |
| Country: China Taxonomical Method(s): Not reported | |
| **Algae:** | **Cyanobacteria:**  *Lyngbya allorgei* (Currently *Phormidium allorgei*)  *Phormidium calcicola*  *Phormidium tenue* (Currently *Leptolyngbya tenuis*) |
| Paper 108 – Williams W, Büdel B, Williams S (2018) | |
| Country: Australia Taxonomical Method(s): Morphology | |
| **Algae:** | **Cyanobacteria:**  *Microcoleus vaginatus*  *Nostoc commune*  *Nostoc* sp.  *Oscillatoria* sp.  *Phormidium* sp.  *Porphyrosiphon* sp.  *Schizothrix* sp.  *Scytonema* sp.  *Symploca* sp.  *Symplocastrum* sp. |
| Paper 109 – Williams W, Chilton A, Schneemilch M *et al.* (2019) | |
| Country: Australia Taxonomical Method(s): Morphology and Molecular Biology | |
| **Algae:** | **Cyanobacteria:**  *Brasilonema*  *Chroococcidiopsis*  *Chroococcus*  *Gloeocapsa*  *Leptolyngbya*  *Microcoleus paludosus*  *Nostoc*  *Porphyrosiphon*  *Schizothrix*  *Scytonema*  *Symploca*  *Symplocastrum* |
| Paper 110 – Wu Y, Li X, Hasi-Eerdun *et al.* (2020) | |
| Country: China Taxonomical Method(s): Not reported | |
| **Algae:** | **Cyanobacteria:**  *Lyngbya* spp.  *Microcoleus vaginatus*  *Oscillatoria* spp. |
| Paper 111 – Xiao B, Sun F, Hu K *et al.* (2019) | |
| Country: China Taxonomical Method(s): Not reported | |
| **Algae:** | **Cyanobacteria:**  *Lyngbya allorgei* (Currently *Phormidium allorgei*)  *Phormidium angustissimum* (Currently *Leptolyngbya angustissima*) |

| Paper 112 – Yang X, Xu M, Zhao Y *et al.* (2019) | |
| --- | --- |
| Country: China Taxonomical Method(s): Not reported | |
| **Algae:** | **Cyanobacteria:**  *Lyngbya allorgei (Currently Phormidium allorgei)*  *Phormidium calcicola*  *Phormidium tenue* (Currently *Leptolyngbya tenuis*) |
| Paper 113 – Yeager CM, Kuske CR, Carney TD *et al.* (2012) | |
| Country: United States (USA) Taxonomical Method(s): Molecular Biology | |
| **Algae:**: | **Cyanobacteria:**  *Microcoleus vaginatus*  *Nostoc commune*  *Scytonema hyalinum*  *Spirirestis rafaelensis* |
| Paper 114 – Zaady E, Katra I, Yizhaq H *et al.* (2014) | |
| Country: Israel Taxonomical Method(s): Not reported | |
| **Algae:** | **Cyanobacteria:**  *Calothrix parietina*  *Microcoleus sociatus*  *Nostoc* sp. |
| Paper 115 – Zhang Y, Duan P, Zhang P *et al.* (2018) | |
| Country: China Taxonomical Method(s): Morphology | |
| **Algae:** | **Cyanobacteria:**  *Aphanizomenon* sp.  *Lyngbya* sp.  *Microcoleus vaginatus*  *Nostoc* sp.  *Oscillatoria* sp.  *Phormidium* sp.  *Phormidium tenue* (Currently *Leptolyngbya tenuis*)  *Scytonema* sp. |
| Paper 116 – Zhang X, Li J, Liu J *et al.* (2021) | |
| Country: China Taxonomical Method(s): Molecular Biology | |
| **Algae:** | **Cyanobacteria:**  *Aliterella*  *Chroococcidiopsis*  *Leptolyngbya*  *Nostoc*  *Scytonema*  *Trichocoleus* |

| Paper 117 – Zhao Y, Xu M, Belnap J (2010) | |
| --- | --- |
| Country: China Taxonomical Method(s): Morphology | |
| **Algae:**  *Chlorococcum* sp.  *Chlorella vulgaris*  *Pinnularia* sp.  *Tribonema bombycinum* | **Cyanobacteria:**  *Asterocapsa* sp.  *Chroococcus turgidus*  *Gloeocapsa magma* (Currently *Gloeocapsopsis magma*)  *Gloeocapsa punctata*  *Gloeocapsa* sp.  *Nostoc calcicola*  *Nostoc commune*  *Nostoc punctiforme*  *Nostoc* sp.  *Oscillatoria* sp.  *Petalonema alatum* |
| Paper 118 – Zhao K, Zhang B, Li J *et al.* (2021) | |
| Country: China Taxonomical Method(s): Molecular Biology | |
| **Algae:**  *Diplosphaera* sp.  Makinoelloideae sp.  *Trebouxia* sp. | **Cyanobacteria:** |
| Paper 119 – Zhao Y, Jia RL, Wang J (2019) | |
| Country: China Taxonomical Method(s): Morphology | |
| **Algae:** | **Cyanobacteria:**  *Anabaena* sp.  *Nostoc* sp.  *Phormidium* sp.  *Scytonema* sp.  *Tolypothrix* sp. |
| Paper 120 – Zhou XB, Zhang YM, Yin BF (2016) | |
| Country: China Taxonomical Method(s): Not reported | |
| **Algae:** | **Cyanobacteria:**  *Chroococcus turgidus var. solitarius Microcoleus paludosus*  *Microcoleus vaginatus*  *Nostoc* sp.  *Xenococcus lyngbyae* |
| Paper 121 – Zubiaga EB, Álvarez GH (2018) | |
| Country: Mexico Taxonomical Method(s): Morphology | |
| **Algae:** | **Cyanobacteria:**  *Chroococcidiopsis* sp.  *Chroococcus* sp.  *Nostoc* sp.  *Schyzothrix* sp.  *Scytonema javanicum* |

REFERENCES

Aanderud ZT, Bahr J, Robinson DM *et al*. The Burning of Biocrusts Facilitates the Emergence of a Bare Soil Community of Poorly-Connected Chemoheterotrophic Bacteria With Depressed Ecosystem Services. *Front Ecol Evol* 2019;7:467.

Abed RMM, Tamm A, Hassenrück C *et al.* Habitat-dependent composition of bacterial and fungal communities in biological soil crusts from Oman. *Sci Rep* 2019;9:6468.

Antoninka A, Bowker MA, Chuckran P *et al.* Maximizing establishment and survivorship of field-collected and greenhouse-cultivated biocrusts in a semi-cold desert. *Plant Soil* 2018;429: 213–225.

Antoninka A, Bowker MA, Reed SC *et al*. Production of greenhouse-grown biocrust mosses and associated cyanobacteria to rehabilitate dryland soil function. *Restor Ecol* 2016;24:324-335.

Ayuso SV, Giraldo-Silva A, Barger NN *et al*. Microbial inoculum production for biocrust restoration: testing the effects of a common substrate versus native soils on yield and community composition. *Restor Ecol* 2020;28:S194-S202.

Ayuso VS, Giraldo Silva A, Nelson C *et al*. Microbial nursery production of high-quality biological soil crust biomass for restoration of degraded dryland soils. *Appl Environ Microbiol* 2017;83 (3):e02179–e2216.

Bailet B, Bouchez A, Franc A *et al*. Molecular versus morphological data for benthic diatoms biomonitoring in Northern Europe freshwater and consequences for ecological status. *Metabarcoding Metagen* 2019;3:e34002.

Bao T, Zhao Y, Gao HJLL *et al*. Moss-dominated biocrusts improve the structural diversity of underlying soil microbial communities by increasing soil stability and fertility in the Loess Plateau region of China. *Eur J Soil Biol* 2019;95:103120.

Bao T, Zhao Y, Yang X *et al*. Effects of disturbance on soil microbial abundance in biological soil crusts on the Loess Plateau, China. *J Arid Environ* 2019;163:59-67.

Bastida F, Jehmlich N, Ondoño S *et al*. Characterization of the microbial community in biological soil crusts dominated by Fulgensia desertorum (Tomin) Poelt and Squamarina cartilaginea (With.) P. James and in the underlying soil. *Soil Biol & Biochem* 2014;76:70–79.

Becerra-Absalón I, Johansen JR, Muñoz-Martín MA *et al*. Chroakolemma gen. nov. (Leptolyngbyaceae, Cyanobacteria) from soil biocrusts in the semi-desert Central Region of Mexico. *Phytotaxa* 2018;367:201–218.

Becerra-Absalón I, Muñoz-Martín MÁ, Montejano G *et al*. Differences in the Cyanobacterial Community Composition of Biocrusts From the Drylands of Central Mexico. Are There Endemic Species? Fron microbiol 2019;10;937.

Belnap J, Büdel B. Biological Soil Crusts as Soil Stabilizers. In: Weber B., Büdel B., Belnap J. (eds) Biological Soil Crusts: An Organizing Principle in Drylands. Ecological Studies (Analysis and Synthesis). *Springer* 2016;226.

Bengtsson MM, Wagner K, Schwab C *et al*. Light availability impacts structure and function of phototrophic stream biofilms across domains and trophic levels. *Mol Ecol* 2018;27(14):2913-2925.

Borchhardt N, Baum C, Thiem D *et al*. Soil microbial phosphorus turnover and identity of algae and fungi in biological soil crusts along a transect in a glacier foreland. *Eur J Soil Biol* 2019;91:9-17.

Bowker MA, Antoninka AJ, Chuckran PF. Improving field success of biocrust rehabilitation materials: hardening the organisms or softening the environment? *Restor Ecol* 2020;28:S177-S186.

Büdel B, Williams WJ, Reichenberger H. Annual net primary productivity of a cyanobacteria-dominated biological soil crust in the Gulf Savannah, Queensland, Australia. *Biogeosciences* 2018;15:491–505.

Caesar J, Tamm A, Ruckteschler N *et al*. Revisiting chlorophyll extraction methods in biological soil crusts – methodology for determination of chlorophyll a and chlorophyll a + b as compared to previous methods. *Biogeosciences* 2018;15:1415–1424.

Cano-Díaz C, Mateo P, Muñoz-Martín MA *et al*. Diversity of biocrust-forming cyanobacteria in a semiarid gypsiferous site from Central Spain. *J Arid Environ* 2018;151:83-89.

Cantón Y, Chamizo S, Rodriguez-Caballero E *et al*. Water Regulation in Cyanobacterial Biocrusts from Drylands: Negative Impacts of Anthropogenic Disturbance. *Water* 2020;12:720.

Castillo-Monroy AP, Benítez A, Reyes-Bueno F et al. Biocrust structure responds to soil variables along a tropical scrubland elevation gradient*. J Arid Environ* 2016;124:31-38.

Chamizo S, Adessi A, Certini G *et* al. Cyanobacteria inoculation as a potential tool for stabilization of burned soils. *Restor Ecol* 2020;28:S106-S114.

Chamizo S, Adessi A, Torzillo G *et* al. Exopolysaccharide Features Influence Growth Success in Biocrust-forming Cyanobacteria, Moving From Liquid Culture to Sand Microcosms. *Front Microbiol* 2020;11:568224.

Chamizo S, Mugnai G, Rossi F *et al*. Cyanobacteria Inoculation Improves Soil Stability and Fertility on Different Textured Soils: Gaining Insights for Applicability in Soil Restoration. *Front Environ Sci* 2018;6:49.

Chilton AM, Neilan BA, Eldridge DJ. Biocrust morphology is linked to marked differences in microbial community composition. *Plant Soil* 2018;429:65–75.

Chua M, Erickson TE, Merritt DJ *et al*. Bio-priming seeds with cyanobacteria: effects on native plant growth and soil properties. *Restor Ecol* 2020;28:S168-S176.

Condon LA, Pietrasiak N, Rosentreter R *et* al. Passive restoration of vegetation and biological soil crusts following 80 years of exclusion from grazing across the Great Basin. *Restor Ecol* 2020;28:S75-S85.

Couradeau E, Giraldo-Silva A, De Martini F *et al.* Spatial segregation of the biological soil crust microbiome around its foundational cyanobacterium, *Microcoleus vaginatus*, and the formation of a nitrogen-fixing cyanosphere. *Microbiome* 2019;7:55 (2019). DOI: https://doi.org/10.1186/s40168-019-0661-2

Couradeau E, Karaoz U, Lim H *et al.* Bacteria increase arid-land soil surface temperature through the production of sunscreens. *Nat Commun* 2016;7:10373.

Dettweiler-Robinson E, Sinsabaugh RL, Rudgers JA. Biocrusts benefit from plant removal. *Am J Bot* 2018;105(7):1133-1141.

Doherty KD, Bowker MA, Antoninka AJ *et al.* Biocrust moss populations differ in growth rates, stress response, and microbial associates. *Plant Soil* 2018;429:187–198.

Dojani S, Kauff F, Weber B *et al.* Genotypic and Phenotypic Diversity of Cyanobacteria in Biological Soil Crusts of the Succulent Karoo and Nama Karoo of Southern Africa. *Microb Ecol* 2014;67:286–301.

Dulić T, Meriluoto J, Malešević TP *et al*. Cyanobacterial diversity and toxicity of biocrusts from the Caspian Lowland loess deposits, North Iran. *Quat Int* 2017;429:74-85.

Fattahi SM, Soroush A, Huang N *et al*. Laboratory study on biophysicochemical improvement of desert sand. *CATENA* 2020;190:104531.

Fattahi SM, Soroush A, Huang N. Wind erosion control using inoculation of aeolian sand with cyanobacteria. *Land Degrad Develop* 2020;31:2104–2116.

Fernandes VMC, Machado de Lima NM, Roush D *et al*. Exposure to predicted precipitation patterns decreases population size and alters community structure of cyanobacteria in biological soil crusts from the Chihuahuan Desert. *Environ Microbiol* 2018;20(1):259-269.

Fischer T. PCA-based supervised identification of biological soil crusts in multispectral images. *MethodsX* 2019;6:764-772.

Flechtner VR, Johansen JR, Belnap J. The Biological Soil Crusts of the San Nicolas Island: Enigmatic Algae from a Geographically Isolated Ecosystem *West N Am Nat* 2008;68(4):405-436.

Forster RM, Créach K, Sabbe W *et al*. Biodiversity–ecosystem function relationship in microphytobenthic diatoms of the Westerschelde estuary. *Mar Ecol Prog Ser* 2006;311:191-201.

Gao L, Bowker MA, Sun H. Linkages between biocrust development and water erosion and implications for erosion model implementation. *Geoderma* 2020;357(1):113973.

Gao L, Sun H, Xu M *et al.* Biocrusts resist runoff erosion through direct physical protection and indirect modification of soil properties. *J Soils Sediments* 2020;20**:**133–142.

García-Meza JV, Carrillo-Chávez A, Morton-Bermea O. Sequential extractions on mine tailings samples after and before bioassays: implications on the speciation of metals during microbial re-colonization. Environ Geol 2006;49:437-448.

Giraldo-Silva A, Fernandes V, Bethany J *et al*. Niche Partitioning with Temperature among Heterocystous Cyanobacteria (*Scytonema* spp., *Nostoc* spp., and *Tolypothrix* spp.) from Biological Soil Crusts. *Microorganisms* 2020;*8*(3):396.

Giraldo-Silva A, Nelson C, Barger NN *et* al. Nursing biocrusts: isolation, cultivation, and fitness test of indigenous cyanobacteria. *Restor Ecol* 2019;27: 793-803.

Giraldo-Silva A, Nelson C, Penfold C *et* al. Effect of preconditioning to the soil environment on the performance of 20 cyanobacterial strains used as inoculum for biocrust restoration. *Restor Ecol* 2020;28:S187-S193.

Grishkan I, Kidron GJ. Biocrust-inhabiting cultured microfungi along a dune catena in the western Negev Desert, Israel. *Eur J Soil Biol* 2013;56:107-114.

Gypser S, Herppich WB, Fischer T *et al*. Photosynthetic characteristics and their spatial variance on biological soil crusts covering initial soils of post-mining sites in Lower Lusatia, NE Germany. Flora - Morphology, Distribution, Funct Ecol 2016;220:103-116.

Gypser S, Veste M, Fischer T *et al*. Infiltration and water retention of biological soil crusts on reclaimed soils of former open-cast lignite mining sites in Brandenburg, north-east Germany. *J Hydrol Hydromech* 2016;64(1):1-11.

Hakkoum Z, Minaoui F, Douma M *et al*. Diversity and spatial distribution of soil cyanobacteria along an altitudinal gradient in Marrakesh area (Morocco). *Appl Ecol Env Res* 2020;18(4):5527–5545.

Hashim ZE, Al-Madhhachi AT, Alzubaidi LA. Behavior of soil erodibility parameters due to biological soil crusts using jet erosion tests. *Ecol Eng* 2020;153:105903.

Jia R, Teng J, Chen M *et al*. The differential effects of sand burial on CO2, CH4, and N2O fluxes from desert biocrust-covered soils in the Tengger Desert, China. *CATENA* 2018;160:252-260.

Karaoz U, Couradeau E, Rocha UM *et al*. Large Blooms of *Bacillales* (*Firmicutes*) Underlie the Response to Wetting of Cyanobacterial Biocrusts at Various Stages of Maturity. *mBio* 2018;9(2):e01366-16.

Kheirfam H, Asadzadeh F. Stabilizing sand from dried-up lakebeds against wind erosion by accelerating biological soil crust development. *Eur J Soil Biol* 2020;98:103189.

Kheirfam H, Roohi M. Accelerating the formation of biological soil crusts in the newly dried-up lakebeds using the inoculation-based technique. *Sci Total Environ* 2020;706:136036.

Kheirfam H. Increasing soil potential for carbon sequestration using microbes from biological soil crusts. *J Arid Environ* 2020;172:104022.

Kidron GJ, Xiao B, Benenson I. Data variability or paradigm shift? Slow versus fast recovery of biological soil crusts-a review. *Sci Total Environ* 2020;721:137683.

Kremer B, Kaźmierczak J, Środoń J. Cyanobacterial-algal crusts from Late Ediacaran paleosols of the East European Craton. Precambrian Res 2018;305:236-246.

Kuske C, Yeager C, Johnson S *et al.* Response and resilience of soil biocrust bacterial communities to chronic physical disturbance in arid shrublands. *ISME J* 2012;6:886–897

Ladislav H, Brinkmann N, Mohr KI *et al*. Diversity of microscopic green algae (Chlorophyta) in calcifying biofilms of two karstic streams in Germany.*Geomicrobiol J* 2015;32:275-290.

Lan S, Thomas AD, Tooth S *et al*. Effects of vegetation on bacterial communities, carbon and nitrogen in dryland soil surfaces: implications for shrub encroachment in the southwest Kalahari. *Sci Total Environ* 2021;764:142847.

Li H, Li R, Rossi F *et al*. Differentiation of microbial activity and functional diversity between various biocrust elements in a heterogeneous crustal community. *CATENA* 2016;147:138-145.

Li J, Jin X, Zhang X *et al*. Comparative metagenomics of two distinct biological soil crusts in the Tengger Desert, China. *Soil Biol Biochem* 2020;140:107637.

Lorite J, Agea D, García-Robles H *et* al. Plant recovery techniques do not ensure biological soil-crust recovery after gypsum quarrying: a call for active restoration. *Restor Ecol* 2020;28:S86-S95.

Machado-de-Lima NM, Branco LHZ. Biological soil crusts: new genera and species of Cyanobacteria from Brazilian semi-arid regions. *Phytotaxa* 2020;470(4):263 – 281.

Machado-de-Lima NM, Fernandes VMC, Roush D *et al*. The Compositionally Distinct Cyanobacterial Biocrusts From Brazilian Savanna and Their Environmental Drivers of Community Diversity. *Front Microbiol* 2019;10:2798.

Machado-de-Lima NM, Muñoz-Rojas M, Vázquez-Campos X *et al*. Biocrust cyanobacterial composition, diversity, and environmental drivers in two contrasting climatic regions in Brazil. *Geoderma* 2021;386:114914.

Maier S, Tamm A, Wu D *et al.* Photoautotrophic organisms control microbial abundance, diversity, and physiology in different types of biological soil crusts. *ISME J* 2018;12:1032–1046.

Moreira-Grez B, Tam K, Cross AT *et al*. The Bacterial Microbiome Associated With Arid Biocrusts and the Biogeochemical Influence of Biocrusts Upon the Underlying Soil. *Front Microbiol* 2019;10:2143.

Moya P, Molins A, Chiva S *et al*. Symbiotic microalgal diversity within lichenicolous lichens and crustose hosts on Iberian Peninsula gypsum biocrusts. *Sci Rep* 2020;10(1);14060.

Mugnai G, Rossi F, Chamizo S *et al*. The role of grain size and inoculum amount on biocrust formation by *Leptolyngbya ohadii*. *CATENA* 2020;184:104248.

Mugnai G, Rossi F, Felde VJMNL *et al.* Development of the polysaccharidic matrix in biocrusts induced by a cyanobacterium inoculated in sand microcosms. *Biol Fertil Soils* 2018;54:27–40.

Muñoz-Martín MÁ, Becerra-Absalón I, Perona E *et al*. Cyanobacterial biocrust diversity in Mediterranean ecosystems along a latitudinal and climatic gradient. *New Phytol* 2019;221(1):123-141.

Muñoz-Rojas M, Chilton A, Liyanage G *et al*. Effects of indigenous soil cyanobacteria on seed germination and seedling growth of arid species used in restoration. *Springer* 2018;*429*(1-2):91–100.

Muñoz-Rojas M, Román JR, Roncero-Ramos B *et al*. Cyanobacteria inoculation enhances carbon sequestration in soil substrates used in dryland restoration. *Sci Total Environ* 2018;636:1149-1154.

Nejidat A, Potrafka RM, Zaady E. Successional biocrust stages on dead shrub soil mounds after severe drought: Effect of micro-geomorphology on microbial community structure and ecosystem recovery. *Soil Biol Biochem* 2016;103:213-220.

Nelson C, Giraldo-Silva A, Garcia-Pichel F. A Fog-Irrigated Soil Substrate System Unifies and Optimizes Cyanobacterial Biocrust Inoculum Production. *Appl Environ Microbiol* 2020;86(13):e00624-20.

Nowicka-Krawczyk P, Żelazna-Wieczorek J, Otlewska A *et al*. Diversity of an aerial phototrophic coating of historic buildings in the former Auschwitz II-Birkenau concentration camp. *Sci Total Environ* 2014;493:116-123.

Ochoa-Huesco R, Mondragon-Cortés T, Concostrina-Zubiri L *et al*. Nitrogen deposition reduces the cover of biocrust-forming lichens and soil pigment content in a semiarid Mediterranean shrubland. *Environ Sci Pollut Res* 2017;24: 26172-26184.

Ouyang H, Hu C. Insight into climate change from the carbon exchange of biocrusts utilizing non-rainfall water. *Sci Rep* 2017;7:2573.

Panigada C, Tagliabue G, Zaady E *et al*. A new approach for biocrust and vegetation monitoring in drylands using multi-temporal Sentinel-2 images. *Prog Phys Geogr* 2019;43(4):496–520.

Pombubpa N, Kurbessoian T, Stajich JE *et al*. Exploring the Microbial Diversity in Biological Soil Crusts at Joshua Tree National Park. *Joshua Tree Science – National Park Service* 2020.

Pombubpa N, Pietrasiak N, Ley PD *et al*. Insights into dryland biocrust microbiome: geography, soil depth and crust type affect biocrust microbial communities and networks in Mojave Desert, USA. *FEMS Microbiol Ecol* 2020;96(9):fiaa125.

Pushkareva E, Baumann K, Van AT *et al*. Diversity of microbial phototrophs and heterotrophs in Icelandic biocrusts and their role in phosphorus-rich Andosols. *Geoderma* 2021;386:114905.

Rachel K. Thiet, R.E.J. Boerner, Moria Nagy, Richard Jardine. The effect of biological soil crusts on throughput of rainwater and N into Lake Michigan sand dune soils. Plant and Soil 278:235-251 (2005).

Rippin M, Borchhardt N, Karsten U and Becker B. (2019) **Cold Acclimation Improves the Desiccation Stress Resilience of Polar Strains of *Klebsormidium* (Streptophyta)**. *Front. Microbiol.* 10:1730. doi: 10.3389/fmicb.2019.01730.

Rippin M, Lange S, Sausen N *et al*. Biodiversity of biological soil crusts from the Polar Regions revealed by metabarcoding. *FEMS microbiol ecol* 2018;94:fiy036.

Rocha F, Lucas-Borja, ME, Pereira P *et al*. Cyanobacteria as a Nature-Based Biotechnological Tool for Restoring Salt-Affected Soils. *Agronomy* 2020;10:1321.

Román JR, Chilton AM, Cantón Y *et al*. Assessing the viability of cyanobacteria pellets for application in arid land restoration. *J Environ Manage* 2020;270:110795.

Román JR, Roncero-Ramos B, Chamizo S *et al*. Restoring soil functions by means of cyanobacteria inoculation: Importance of soil conditions and species selection. *Land Degrad Dev* 2018;29:3184– 3193.

Romero AN, Moratta MH, Vento B *et* al. Variations in the coverage of biological soil crusts along a gradient of aridity in the center-west of argentina. *bioRxiv* 2019. DOI: https://doi.org/10.1101/725986

Roncero-Ramos B, Muñoz-Martín MA, Cantón Y *et al*. Land degradation effects on composition of pioneering soil communities: An alternative successional sequence for dryland cyanobacterial biocrusts. *Soil Biol Biochem* 2020;146:107824.

Roncero-Ramos B, Muñoz-Martín MÁ, Chamizo S *et al*. Polyphasic evaluation of key cyanobacteria in biocrusts from the most arid region in Europe. *PeerJ* 2019;7:e6169.

Samolov E, Baumann K, Büdel B *et al*. Biodiversity of Algae and Cyanobacteria in Biological Soil Crusts Collected Along a Climatic Gradient in Chile Using an Integrative Approach. *Microorganisms* 2020;*8*:1047.

Schulz K, Mikhailyuk T, Dreßler M *et al*. Biological Soil Crusts from Coastal Dunes at the Baltic Sea: Cyanobacterial and Algal Biodiversity and Related Soil Properties. *Microb Ecol* 2016;71:178–193.

Sepehr A, Hasssanzadeh M, Rodriguez-Caballero E. The protective role of cyanobacteria on soil stability in two Aridisols in northeastern Iran. *Geoderma Reg* 2019;16:e00201.

Sommer V, Karsten U, Glaser K. Halophilic Algal Communities in Biological Soil Crusts Isolated From Potash Tailings Pile Areas. *Front Ecol Evol* 2020;8:46.

Sommer V, Mikhailyuk T, Glaser K *et al*. Uncovering Unique Green Algae and Cyanobacteria Isolated from Biocrusts in Highly Saline Potash Tailing Pile Habitats, Using an Integrative Approach. *Microorganisms* 2020;*8*:1667.

Sorochkina K, Ayuso SV, Garcia-Pichel F. Establishing rates of lateral expansion of cyanobacterial biological soil crusts for optimal restoration. *Plant Soil* 2018;*429*(1-2):199-211.

Steven B, Belnap J and Kuske CR. Chronic Physical Disturbance Substantially Alters the Response of Biological Soil Crusts to a Wetting Pulse, as Characterized by Metatranscriptomic Sequencing. *Front Microbiol* 2018;9:2382.

Steven B, Gallegos-Graves LV, Belnap J *et al*. Dryland soil microbial communities display spatial biogeographic patterns associated with soil depth and soil parent material. *FEMS Microbiol Ecol* 2013;86(1):101-13.

Steven B, Gallegos-Graves LV, Yeager CM *et al*. Dryland biological soil crust cyanobacteria show unexpected decreases in abundance under long-term elevated CO2. *Environ Microbiol* 2012;14(12):3247-58.

Steven B, Yeager C, Belnap J *et al*. Common and distinguishing features of the bacterial and fungal communities in biological soil crusts and shrub root zone soils. *Soil Biol Biochem* 2014;69:302-312.

Stricker E, Crain G, Rudgers J *et al*. What Could Explain δ 13 C Signatures in Biocrust Cyanobacteria of Drylands?. *Microb ecol* 2021;81:134-145.

Szyja M, Menezes AGS, Oliveira FDA *et al*. Neglected but Potent Dry Forest Players: Ecological Role and Ecosystem Service Provision of Biological Soil Crusts in the Human-Modified Caatinga. *Front Ecol Evol* 2019;7:482.

Tamm A, Caesar J, Kunz N *et al.* Ecophysiological properties of three biological soil crust types and their photoautotrophs from the Succulent Karoo, South Africa. *Plant Soil* 2018;429:127–146.

Vinoth M, Sivasankari S, Ahamed A *et al*. Biological soil crust (BSC) is an effective biofertilizer on *Vigna mungo* (L.). *Saudi J Biol Sci* 2020;27(9):2325–2332.

Wang L, Zhang G, Zhu L *et* al. Biocrust wetting induced change in soil surface roughness as influenced by biocrust type, coverage and wetting patterns. Geoderma 2017;306(15):1–9.

Williams W, Büdel B, Williams S. Wet season cyanobacterial N enrichment highly correlated with species richness and *Nostoc* in the northern Australian savannah, *Biogeosciences* 2018;15:2149–2159.

Williams W, Chilton A, Schneemilch M *et al*. Microbial biobanking – cyanobacteria-rich topsoil facilitates mine rehabilitation. *Biogeosciences* 2019;16:2189–2204.

Wu Y, Li X, Hasi-Eerdun *et al*. Surface roughness response of biocrust-covered soil to mimicked sheep trampling in the Mu Us sandy Land, northern China. *Geoderma* 2020;363:114146.

Xiao B, Sun F, Hu K *et al*. Biocrusts reduce surface soil infiltrability and impede soil water infiltration under tension and ponding conditions in dryland ecosystem. *J Hydrol* 2019;568:792-802.

Yang X, Xu M, Zhao *Y et al*. Moss-dominated biological soil crusts improve stability of soil organic carbon on the Loess Plateau, China. *Plant Soil Environ* 2019;65:104-109.

Yeager CM, Kuske CR, Carney TD *et al*. Response of biological soil crust diazotrophs to season, altered summer precipitation, and year-round increased temperature in an arid grassland of the Colorado Plateau, USA. *Front Microbio* 2012. 3:358. doi:10.3389/fmicb.2012.00358.

Zaady E, Katra I, Yizhaq H. Inferring the impact of rainfall gradient on biocrusts’ developmental stage and thus on soil physical structures in sand dunes. *Aeolian Res* 2014;13:81-89.

Zhang X, Li J, Liu J *et al*. Temporal shifts in cyanobacterial diversity and their relationships to different types of biological soil crust in the southeastern Tengger Desert. *Rhizosphere* 2021;17:100322.

Zhang Y, Duan P, Zhang P *et al.* Variations in cyanobacterial and algal communities and soil characteristics under biocrust development under similar environmental conditions. *Plant Soil* 2017;429:241–251.

Zhao K, Zhang B, Li J *et al*. The autotrophic community across developmental stages of biocrusts in the Gurbantunggut Desert. *Geoderma* 2021;388:114927.

Zhao Y, Jia RL, Wang J. Towards stopping land degradation in drylands: Water-saving techniques for cultivating biocrusts in situ. *Land Degrad Dev* 2019;30:2336– 2346.

Zhao Y, Xu M, Belnap J. Potential nitrogen fixation activity of different aged biological soil crusts from rehabilitated grasslands of the hilly Loess Plateau, China. *J Arid Environ* 2010;74:1186-1191.

Zhou XB, Zhang YM, Yin BF. Divergence in physiological responses between cyanobacterial and lichen crusts to a gradient of simulated nitrogen deposition. *Plant Soil* 2016;399:121–134.

Zubiaga EB, Álvarez GH. Infiltración en biocostras en una región semiárida del centro de México. *Terra Latinoamericana* 2018;36:337-343. Arp G, Bisset A, Brinkmann N *et al*. Tufa-forming biofilms of German karstwater streams: Microorganisms, exopolymers, hydrochemistry and calcification. *Geol Soc Spec Publ* 2010;336:83-118.
