# Supplementary material for: Organizing a global list of cyanobacteria and algae from soil biocrusts evidenced great geographic and taxonomic gaps
Source: FEMS Microbiol Ecol. 2024 May 30;100(7):fiae086. doi: 10.1093/femsec/fiae086 (PMC11221558; doi:10.1093/femsec/fiae086)
Supplement: fiae086_Supplemental_Files [file fiae086_supplemental_files.zip › Supplementary Material S2.docx]

**Supplementary Material S2: Characteristics of the environments where each Cyanobacteria taxon was observed.**

| **Region** | **Substrate** | **Climate** | **Species** | **References** |
| --- | --- | --- | --- | --- |
| Temperate | Rock | Semiarid | *Chroococcidiopsis* sp.; *Leptolyngbya frigida* (Currently *Stenomitos frigidus*); *Leptolyngbya* sp.; *Microcoleus* sp.; *Microcoleus steenstrupii*; *Microcoleus vaginatus*; *Nostoc commune*; *Phormidium* sp.; *Scytonema* sp.; *Tolypothrix distorta* | Román *et al*. 2018*; Roncero-Ramos *et al*. 2019*; Cantón *et al*. 2020*; Roncero-Ramos *et al*. 2020* |
|  |  | Semiarid to subhumid | *Leptolyngbya foveolarum*; *Leptolyngbya* sp. | Hakkoum *et al*. 2020 |
|  |  | Continental Mediterranean | *Microcoleus* sp.; *Nostoc* sp.; *Schizothrix* sp. | Lorite *et al*. 2020* |
|  |  | Humid | *Chroococcus* sp.; *Leptolyngbya foveolarum*; *Leptolyngbya* sp. | Hakkoum *et al*. 2020 |
|  |  | No information | *Chroococcus* sp.; *Gloeocapsa* sp.; *Leptolyngbya foveolarum*; *Leptolyngbya frigida* (Currently *Stenomitos frigidus*); *Microcoleus vaginatus*; *Nostoc commune*; *Nostoc punctiforme*; *Nostoc* sp.; *Tolypothrix* sp. | Nowicka-Krawczyk *et al*. 2014 |
|  | Soil | Hyperarid | *Microcoleus vaginatus*; *Nostoc commune*; *Nostoc* sp. | Romero *et al*. 2020 |
|  |  | Arid | *Anabaena* sp.; *Calothrix* sp.; *Chroococcidiopsis* sp.; *Chroococcus* sp.; *Leptolyngbya* sp.; *Lyngbya* sp.; *Microcoleus paludosus*; *Microcoleus* sp.; *Microcoleus steenstrupii*; *Microcoleus vaginatus*; *Nostoc commune*; *Nostoc* sp.; *Oscillatoria* sp.; *Phormidium* sp,; *Schizothrix* sp.; *Scytonema* sp.; *Synechococcus* sp; *Tolypothrix distorta*; *Tolypothrix* sp.; *Trichocoleus* sp. | Kuske *et al*. 2012; Steven *et al*. 2012*; Steven *et al*. 2013; Zhao *et al*. 2019*; Ayuso *et al*. 2020; Condon *et al*. 2020; Flechtner *et* al. 2009*; Grishkan, Kidron 2013; Zaady *et al*. 2014; Dojani *et al*. 2014; Román *et al*. 2018; Sorochkina *et al*. 2018*; Panigada *et al*. 2019; Hakkoum *et* al. 2020; Romero *et al*. 2020; Nelson *et al*. 2020; Samolov *et al*. 2020; Zhou *et al*. 2016; Li *et al*. 2016; Antoninka *et al*. 2018*; Jia *et al*. 2018*; Fernandes *et al*. 2018*; Aanderud *et al*. 2018*; Li *et al*. 2020; Kidron *et al.* 2020*; Stricker *et al*. 2021 |
|  |  | Semiarid | *Anabaena* sp.; *Chroococcidiopsis* sp.; *Chroococcus* sp.; *Gloeocapsa* sp.; *Leptolyngbya frigida* (Currently *Stenomitos frigidus*); *Leptolyngbya* sp.; *Lyngbya* sp.; *Microcoleus* sp.; *Microcoleus steenstrupii*; *Microcoleus vaginatus*; *Nostoc commune*; *Nostoc* sp.; *Nostoc paludosum*; *Oscillatoria* sp.; *Phormidium* sp.; *Phormidium tenue* (currently *Leptolyngbya tenuis*)*;* *Schizothrix* sp.; *Scytonema* sp.; *Synechococcus* sp.; *Tolypothrix distorta*; *Tolypothrix* sp.; *Trichocoleus* sp. | Flechtner *et al*. 2008*; Yeager, *et al*. 2012; Sepehr et al. 2019; Cano-Díaz *et al*. 2018; Zhao *et al*. 2010; Bastida *et al*. 2014; Zaady *et al*. 2014; Nejidat *et* al. 2016; Wang *et al*. 2017; Dulić *et al*. 2017; Chilton *et al*. 2018*; Moreira-Grez *et al*. 2019; Roncero-Ramos *et al*. 2019; Romero *et al*. 2020; Samolov *et al*. 2020; Bao *et al*. 2019^a^; Bao *et al.* 2019^b^; Yang *et al*. 2019; Tamm *et al*. 2018; Panigada *et al*. 2019; Williams *et al*. 2019; Li *et al*. 2020; Gao *et al*. 2020a; Gao *et al*. 2020b; Wu *et al*. 2020; Kidron *et al.* 2020*; Kheirfam 2020 |
|  |  | Semiarid/  Mediterranean | *Calothrix*; Chroococcidiopsis sp.; *Leptolyngbya frigida* (Currently *Stenomitos frigidus*) *Leptolyngbya* sp.; *Microcoleus steenstrupii*; *Microcoleus vaginatus*; *Microcoleus* sp.; *Nostoc* sp.; *Phormidium* sp.; *Schizothrix* sp.; *Scytonema*; *Trichocoleus* sp.; *Tolypothrix* sp. | Muñoz-Martín *et al*. 2019; Ochoa-Hueso *et al*. 2017 |
|  |  | Continental Mediterranean | *Microcoleus* sp.; *Nostoc* sp.; *Schizothrix* sp. | Lorite *et al*. 2020; Samolov *et al*. 2020 |
|  |  | Sub-humid | *Calothrix*; *Chroococcidiopsis*; *Chroococcus*; *Microcoleus vaginatus*; *Nostoc*; *Schizothrix*; *Scytonema*; *Tolypothrix*; *Trichocoleus* | Dulić *et al*. 2017 |
|  |  | Tropical Monsoon | *Microcoleus* sp.; *Nostoc* sp.; *Phormidium tênue* (currently *Leptolyngbya tenuis*); *Scytonema* sp. | Doherty *et al*. 2018; Ouyang, Hu 2016; Yang *et al*. 2019 |
|  |  | Humid | *Chroococcidiopsis* sp.; *Chroococcus* sp.; *Leptolyngbya* sp.; *Lyngbya* sp.; *Microcoleus* sp.; *Microcoleus vaginatus*; *Nostoc commune*; *Nostoc* sp.; *Porphyrosiphon*.; *Schizothrix* sp.; *Scytonema* sp.; *Stigonema* sp.; *Tolypothrix* sp.; | Machado-de-Lima NM *et al*. 2021; Schulz *et al*. 2016; Samolov *et al*. 2020; Hakkoum *et al*. 2020 |
|  |  | Low arctic | *Microcoleus vaginatus*; | Pushkareva *et al*. 2021 |
|  |  | No information | *Chroococcus* sp.; *Gloeocapsa* sp.; *Leptolyngbya foveolarum*; *Leptolyngbya frigida* (Currently *Stenomitos frigidus*); *Microcoleus vaginatus*; *Nostoc commune*; *Nostoc punctiforme*; *Nostoc* sp.; *Tolypothrix* sp. | Nowicka-Krawczyk *et al*. 2014 |
|  | No information | | *Anabaena* sp.; *Calothrix*; *Chroococcidiopsis* spp.; *Leptolyngbya foveolarum*; *Leptolyngbya* spp.; *Lyngbya* sp.; *Microcoleus* sp.; *Microcoleus steenstrupii*; *Microcoleus vaginatus*; *Nostoc* sp.; *Oscillatoria* sp.; *Phormidium* sp.; *Phormidium tenue* (currently *Leptolyngbya tenuis*); *Pseudanabaena* sp.; *Scytonema* sp.; *Synechococcus* spp.; *Tolypothrix sp*.; *Trichocoleus* spp. | Arp *et al*. 2010; Bengtsson et al. 2018; Steven *et al*. 2014; Dojani *et al*. 2014; Antoninka *et al*. 2016; Couradeau *et al*. 2016; Zhang *et al*. 2017; Maier *et al*. 2018; Karaoz *et al*. 2018; Steven *et al*. 2018; Couradeau *et al*. 2019; Giraldo-Silva *et al*. 2019; Fattahi *et al*. 2020^a^; Rocha *et al*. 2020; Fattahi *et al*. 2020^b^; Pombubpa *et al*. 2020^a^; Pombubpa *et al*. 2020^b^; Giraldo-Silva *et al*. 2020^a^; Giraldo-Silva *et al*. 2020^b^ Lan *et al*. 2021; Zhang *et al*. 2021 |
| Tropical | Soil | Arid | *Calothrix* sp.; *Leptolyngbya* sp.; *Microcoleus paludosus*; *Microcoleus* sp.; *Microcoleus vaginatus*; *Nostoc commune*; *Nostoc* sp.; *Phormidium* sp.; *Schizothrix* sp.; *Scytonema* sp.; *Tolypothrix distorta* | Williams *et al*. 2018; Muñoz-Rojas *et al*. 2018^a^*; Román *et al*. 2020; Chua *et al*. 2020* |
|  |  | Semiarid | *Calothrix* sp.; *Chroococcidiopsis* sp.; *Chroococcus* sp.; *Desmococcus*; *Leptolyngbya foveolarum*; *Leptolyngbya frigida* (Currently *Stenomitos frigidus*); *Leptolyngbya* sp.; *Gloeocapsa* sp.; *Microcoleus steenstrupi*; *Microcoleus vaginatus*; *Nostoc commune*; *Nostoc* sp.; *Oscillatoria* sp.; *Schyzothrix* sp.; *Scytonema* sp.; *Synechococcus* sp; *Tolypothrix distorta*; *Tolypothrix* sp. | Machado-de-Lima NM *et al*. 2021; Zubiaga, Álvarez 2018; Román *et al*. 2020; Muñoz-Rojas *et al*. 2018^b^; Becerra-Absalón *et al*. 2019; Szyja *et al*. 2019; |
|  |  | Tropical dry | *Microcoleus paludosus*; *Microcoleus vaginatus*; *Nostoc commune*; *Nostoc* sp.; *Oscillatoria* sp.; *Phormidium* sp.; *Scytonema* sp. | Castillo-Monroy *et al*. 2016 |
|  |  | Semi-humid | *Calothrix*; *Chroococcidiopsis*; *Chroococcus*; *Nostoc*; *Scytonema*; *Stigonema* | Machado-de-Lima NM *et al*. 2019 |
|  |  | Tropical Monsoon | *Chroococcus sp.; Microcoleus paludosus*; *Microcoleus vaginatus*; *Nostoc commune*; *Nostoc* sp.; *Oscillatoria* sp.; *Phormidium* sp.; *Schizothrix* sp.; *Scytonema* sp. | Williams *et al*. 2018 |
|  | No information | | *Chroococcus*; *Lyngbya* sp.; *Microcoleus*; *Nostoc commune*; *Nostoc* sp.; *Oscillatoria*; *Phormidium*; *Schizothrix*; *Scytonema* sp.; *Synechococcus*; *Tolypothrix* | Büdel *et al*. 2018; Vinoth *et al*. 2020 |
| Polar | Soil |  | *Aphanothece*; *Leptolyngbya*; *Microcoleus*; *Nostoc*; *Oscillatoria*; *Phormidium*; *Pseudanabaena* | Rippin *et al*. 2018 |
| Extreme environments | | | *Anabaena* sp.; *Chloroidium*; *Chroococcidiopsis*; *Gloeocapsa* sp.; *Leptolyngbya* sp.; *Microcoleus vaginatus*; *Nostoc* sp.; *Oscillatoria* sp.; *Phormidium* sp.; *Pseudanabaena* sp. | García-Meza *et al*. 2006; Kremer *et al*. 2018; Kheirfam, Asadzadeh 2020; Kheirfam, Roohi 2020; Sommer *et al*. 2020^a^; Sommer *et al*. 2020^b^ |

* The article does not directly mention climate or habitat, but they can be inferred by other information present in the text.

Aanderud ZT, Bahr J, Robinson DM *et al*. The Burning of Biocrusts Facilitates the Emergence of a Bare Soil Community of Poorly-Connected Chemoheterotrophic Bacteria With Depressed Ecosystem Services. *Front Ecol Evol* 2019;7:467.

Antoninka A, Bowker MA, Chuckran P *et al.* Maximizing establishment and survivorship of field-collected and greenhouse-cultivated biocrusts in a semi-cold desert. *Plant Soil* 2018;429: 213–225.

Antoninka A, Bowker MA, Reed SC *et al*. Production of greenhouse-grown biocrust mosses and associated cyanobacteria to rehabilitate dryland soil function. *Restor Ecol* 2016;24:324-335.

Ayuso SV, Giraldo-Silva A, Barger NN *et al*. Microbial inoculum production for biocrust restoration: testing the effects of a common substrate versus native soils on yield and community composition. *Restor Ecol* 2020;28:S194-S202.

Bao T, Zhao Y, Gao HJLL *et al*. Moss-dominated biocrusts improve the structural diversity of underlying soil microbial communities by increasing soil stability and fertility in the Loess Plateau region of China. *Eur J Soil Biol* 2019^a^;95:103120.

Bao T, Zhao Y, Yang X *et al*. Effects of disturbance on soil microbial abundance in biological soil crusts on the Loess Plateau, China. *J Arid Environ* 2019^b^;163:59-67.

Bastida F, Jehmlich N, Ondoño S *et al*. Characterization of the microbial community in biological soil crusts dominated by *Fulgensia desertorum* (Tomin) Poelt and Squamarina cartilaginea (With.) P. James and in the underlying soil. *Soil Biol & Biochem* 2014;76:70–79.

Becerra-Absalón I, Muñoz-Martín MÁ, Montejano G *et al*. Differences in the Cyanobacterial Community Composition of Biocrusts From the Drylands of Central Mexico. Are There Endemic Species? *Front microbiol* 2019;10;937.

Büdel B, Williams WJ, Reichenberger H. Annual net primary productivity of a cyanobacteria-dominated biological soil crust in the Gulf Savannah, Queensland, Australia. *Biogeosciences* 2018;15:491–505.

Cano-Díaz C, Mateo P, Muñoz-Martín MA *et al*. Diversity of biocrust-forming cyanobacteria in a semiarid gypsiferous site from Central Spain. *J Arid Environ* 2018;151:83-89.

Cantón Y, Chamizo S, Rodriguez-Caballero E *et al*. Water Regulation in Cyanobacterial Biocrusts from Drylands: Negative Impacts of Anthropogenic Disturbance. *Water* 2020;12:720.

Castillo-Monroy AP, Benítez A, Reyes-Bueno F et al. Biocrust structure responds to soil variables along a tropical scrubland elevation gradient*. J Arid Environ* 2016;124:31-38.

Chilton AM, Neilan BA, Eldridge DJ. Biocrust morphology is linked to marked differences in microbial community composition. *Plant Soil* 2018;429:65–75.

Chua M, Erickson TE, Merritt DJ *et al*. Bio-priming seeds with cyanobacteria: effects on native plant growth and soil properties. *Restor Ecol* 2020;28:S168-S176.

Condon LA, Pietrasiak N, Rosentreter R *et* al. Passive restoration of vegetation and biological soil crusts following 80 years of exclusion from grazing across the Great Basin. *Restor Ecol* 2020;28:S75-S85.

Couradeau E, Giraldo-Silva A, De Martini F *et al.* Spatial segregation of the biological soil crust microbiome around its foundational cyanobacterium, *Microcoleus vaginatus*, and the formation of a nitrogen-fixing cyanosphere. *Microbiome* 2019;7:55 (2019). DOI: https://doi.org/10.1186/s40168-019-0661-2

Couradeau E, Karaoz U, Lim H *et al.* Bacteria increase arid-land soil surface temperature through the production of sunscreens. *Nat Commun* 2016;7:10373.

Doherty KD, Bowker MA, Antoninka AJ *et al.* Biocrust moss populations differ in growth rates, stress response, and microbial associates. *Plant Soil* 2018;429:187–198.

Dojani S, Kauff F, Weber B *et al.* Genotypic and phenotypic diversity of cyanobacteria in biological soil crusts of the succulent karoo and nama karoo of Southern Africa. *Microb Ecol* 2014;67:286–301.

Dulić T, Meriluoto J, Malešević TP *et al*. Cyanobacterial diversity and toxicity of biocrusts from the Caspian Lowland loess deposits, North Iran. *Quat Int* 2017;429:74-85.

Fattahi SM, Soroush A, Huang N *et al*. Laboratory study on biophysicochemical improvement of desert sand. *CATENA* 2020^a^;190:104531.

Fattahi SM, Soroush A, Huang N. Wind erosion control using inoculation of aeolian sand with cyanobacteria. *Land Degrad Develop* 2020^b^;31:2104–2116.

Fernandes VMC, Machado-de-Lima NM, Roush D *et al*. Exposure to predicted precipitation patterns decreases population size and alters community structure of cyanobacteria in biological soil crusts from the Chihuahuan Desert. *Environ Microbiol* 2018;20(1):259-269.

Flechtner VR, Johansen JR, Belnap J. The biological soil crusts of the San Nicolas Island: enigmatic algae from a geographically isolated ecosystem. *West N Am Nat* 2008;68(4):405-436.

Gao L, Bowker MA, Sun H. Linkages between biocrust development and water erosion and implications for erosion model implementation. *Geoderma* 2020;357(1):113973.

Gao L, Sun H, Xu M *et al.* Biocrusts resist runoff erosion through direct physical protection and indirect modification of soil properties. *J Soils Sediments* 2020;20**:**133–142.

Giraldo-Silva A, Fernandes V, Bethany J *et al*. Niche Partitioning with Temperature among Heterocystous Cyanobacteria (*Scytonema* spp., *Nostoc* spp., and *Tolypothrix* spp.) from Biological Soil Crusts. *Microorganisms* 2020^a^;8(3):396.

Giraldo-Silva A, Nelson C, Barger NN *et* al. Nursing biocrusts: isolation, cultivation, and fitness test of indigenous cyanobacteria. *Restor Ecol* 2019;27: 793-803.

Giraldo-Silva A, Nelson C, Penfold C *et* al. Effect of preconditioning to the soil environment on the performance of 20 cyanobacterial strains used as inoculum for biocrust restoration. *Restor Ecol* 2020^b^;28:S187-S193.

Grishkan I, Kidron GJ. Biocrust-inhabiting cultured microfungi along a dune catena in the western Negev Desert, Israel. *Eur J Soil Biol* 2013;56:107-114.

Hakkoum Z, Minaoui F, Douma M *et al*. Diversity and spatial distribution of soil cyanobacteria along an altitudinal gradient in Marrakesh area (Morocco). *Appl Ecol Env Res* 2020;18(4):5527–5545.

Jia R, Teng J, Chen M *et al*. The differential effects of sand burial on CO2, CH4, and N2O fluxes from desert biocrust-covered soils in the Tengger Desert, China. *CATENA* 2018;160:252-260.

Karaoz U, Couradeau E, Rocha UM *et al*. Large Blooms of *Bacillales* (*Firmicutes*) Underlie the Response to Wetting of Cyanobacterial Biocrusts at Various Stages of Maturity. *mBio* 2018;9(2):e01366-16.

Kheirfam H, Asadzadeh F. Stabilizing sand from dried-up lakebeds against wind erosion by accelerating biological soil crust development. *Eur J Soil Biol* 2020;98:103189.

Kheirfam H, Roohi M. Accelerating the formation of biological soil crusts in the newly dried-up lakebeds using the inoculation-based technique. *Sci Total Environ* 2020;706:136036.

Kheirfam H. Increasing soil potential for carbon sequestration using microbes from biological soil crusts. *J Arid Environ* 2020;172:104022.

Kidron GJ, Xiao B, Benenson I. Data variability or paradigm shift? Slow versus fast recovery of biological soil crusts-a review. *Sci Total Environ* 2020;721:137683.

Kremer B, Kaźmierczak J, Środoń J. Cyanobacterial-algal crusts from Late Ediacaran paleosols of the East European Craton. Precambrian Res 2018;305:236-246.

Kuske C, Yeager C, Johnson S *et al.* Response and resilience of soil biocrust bacterial communities to chronic physical disturbance in arid shrublands. *ISME J* 2012;6:886–897

Lan S, Thomas AD, Tooth S *et al*. Effects of vegetation on bacterial communities, carbon and nitrogen in dryland soil surfaces: implications for shrub encroachment in the southwest Kalahari. *Sci Total Environ* 2021;764:142847.

Li H, Li R, Rossi F *et al*. Differentiation of microbial activity and functional diversity between various biocrust elements in a heterogeneous crustal community. *CATENA* 2016;147:138-145.

Li J, Jin X, Zhang X *et al*. Comparative metagenomics of two distinct biological soil crusts in the Tengger Desert, China. *Soil Biol Biochem* 2020;140:107637.

Lorite J, Agea D, García-Robles H *et* al. Plant recovery techniques do not ensure biological soil-crust recovery after gypsum quarrying: a call for active restoration. *Restor Ecol* 2020;28:S86-S95.

Machado-de-Lima NM, Fernandes VMC, Roush D *et al*. The Compositionally Distinct Cyanobacterial Biocrusts From Brazilian Savanna and Their Environmental Drivers of Community Diversity. *Front Microbiol* 2019;10:2798.

Machado-de-Lima NM, Muñoz-Rojas M, Vázquez-Campos X *et al*. Biocrust cyanobacterial composition, diversity, and environmental drivers in two contrasting climatic regions in Brazil. *Geoderma* 2021;386:114914.

Maier S, Tamm A, Wu D *et al.* Photoautotrophic organisms control microbial abundance, diversity, and physiology in different types of biological soil crusts. *ISME J* 2018;12:1032–1046.

Moreira-Grez B, Tam K, Cross AT *et al*. The Bacterial Microbiome Associated With Arid Biocrusts and the Biogeochemical Influence of Biocrusts Upon the Underlying Soil. *Front Microbiol* 2019;10:2143.

Muñoz-Martín MÁ, Becerra-Absalón I, Perona E *et al*. Cyanobacterial biocrust diversity in Mediterranean ecosystems along a latitudinal and climatic gradient. *New Phytol* 2019;221(1):123-141.

Muñoz-Rojas M, Chilton A, Liyanage G *et al*. Effects of indigenous soil cyanobacteria on seed germination and seedling growth of arid species used in restoration. *Springer* 2018^a^;*429*(1-2):91–100.

Muñoz-Rojas M, Román JR, Roncero-Ramos B *et al*. Cyanobacteria inoculation enhances carbon sequestration in soil substrates used in dryland restoration. *Sci Total Environ* 2018^b^;636:1149-1154.

Nejidat A, Potrafka RM, Zaady E. Successional biocrust stages on dead shrub soil mounds after severe drought: Effect of micro-geomorphology on microbial community structure and ecosystem recovery. *Soil Biol Biochem* 2016;103:213-220.

Nelson C, Giraldo-Silva A, Garcia-Pichel F. A Fog-Irrigated Soil Substrate System Unifies and Optimizes Cyanobacterial Biocrust Inoculum Production. *Appl Environ Microbiol* 2020;86(13):e00624-20.

Ouyang H, Hu C. Insight into climate change from the carbon exchange of biocrusts utilizing non-rainfall water. *Sci Rep* 2017;7:2573.

Panigada C, Tagliabue G, Zaady E *et al*. A new approach for biocrust and vegetation monitoring in drylands using multi-temporal Sentinel-2 images. *Prog Phys Geogr* 2019;43(4):496–520.

Pombubpa N, Kurbessoian T, Stajich JE *et al*. Exploring the Microbial Diversity in Biological Soil Crusts at Joshua Tree National Park. *Joshua Tree Science – National Park Service* 2020.

Pombubpa N, Pietrasiak N, Ley PD *et al*. Insights into dryland biocrust microbiome: geography, soil depth and crust type affect biocrust microbial communities and networks in Mojave Desert, USA. *FEMS Microbiol Ecol* 2020;96(9):fiaa125.

Pushkareva E, Baumann K, Van AT *et al*. Diversity of microbial phototrophs and heterotrophs in Icelandic biocrusts and their role in phosphorus-rich Andosols. *Geoderma* 2021;386:114905.

Rocha F, Lucas-Borja, ME, Pereira P *et al*. Cyanobacteria as a Nature-Based Biotechnological Tool for Restoring Salt-Affected Soils. *Agronomy* 2020;10:1321.

Román JR, Chilton AM, Cantón Y *et al*. Assessing the viability of cyanobacteria pellets for application in arid land restoration. *J Environ Manage* 2020;270:110795.

Román JR, Roncero-Ramos B, Chamizo S *et al*. Restoring soil functions by means of cyanobacteria inoculation: Importance of soil conditions and species selection. *Land Degrad Dev* 2018;29:3184-3193.

Romero AN, Moratta MH, Vento B *et* al. Variations in the coverage of biological soil crusts along a gradient of aridity in the center-west of Argentina. *Acta Oecol* 2020;109:103671. DOI: https://doi.org/10.1016/j.actao.2020.103671.

Roncero-Ramos B, Muñoz-Martín MA, Cantón Y *et al*. Land degradation effects on composition of pioneering soil communities: An alternative successional sequence for dryland cyanobacterial biocrusts. *Soil Biol Biochem* 2020;146:107824.

Roncero-Ramos B, Muñoz-Martín MÁ, Chamizo S *et al*. Polyphasic evaluation of key cyanobacteria in biocrusts from the most arid region in Europe. *PeerJ* 2019;7:e6169.

Samolov E, Baumann K, Büdel B *et al*. Biodiversity of Algae and Cyanobacteria in Biological Soil Crusts Collected Along a Climatic Gradient in Chile Using an Integrative Approach. *Microorganisms* 2020;*8*:1047.

Schulz K, Mikhailyuk T, Dreßler M *et al*. Biological soil crusts from coastal dunes at the Baltic Sea: cyanobacterial and algal biodiversity and related soil properties. *Microb Ecol* 2016;71:178–193.

Sepehr A, Hasssanzadeh M, Rodriguez-Caballero E. The protective role of cyanobacteria on soil stability in two Aridisols in northeastern Iran. *Geoderma Reg* 2019;16:e00201.

Sommer V, Karsten U, Glaser K. Halophilic Algal Communities in Biological Soil Crusts Isolated From Potash Tailings Pile Areas. *Front Ecol Evol* 2020^a^;8:46.

Sommer V, Mikhailyuk T, Glaser K *et al*. Uncovering Unique Green Algae and Cyanobacteria Isolated from Biocrusts in Highly Saline Potash Tailing Pile Habitats, Using an Integrative Approach. *Microorganisms* 2020^b^;8:1667.

Sorochkina K, Ayuso SV, Garcia-Pichel F. Establishing rates of lateral expansion of cyanobacterial biological soil crusts for optimal restoration. *Plant Soil* 2018;*429*(1-2):199-211.

Steven B, Belnap J and Kuske CR. Chronic physical disturbance substantially alters the response of biological soil crusts to a wetting pulse, as characterized by metatranscriptomic sequencing. *Front Microbiol* 2018;9:2382.

Steven B, Gallegos-Graves LV, Belnap J *et al*. Dryland soil microbial communities display spatial biogeographic patterns associated with soil depth and soil parent material. *FEMS Microbiol Ecol* 2013;86(1):101-13.

Steven B, Gallegos-Graves LV, Yeager CM *et al*. Dryland biological soil crust cyanobacteria show unexpected decreases in abundance under long-term elevated CO2. *Environ Microbiol* 2012;14(12):3247-58.

Steven B, Yeager C, Belnap J *et al*. Common and distinguishing features of the bacterial and fungal communities in biological soil crusts and shrub root zone soils. *Soil Biol Biochem* 2014;69:302-312.

Szyja M, Menezes AGS, Oliveira FDA *et al*. Neglected but potent dry forest players: ecological role and ecosystem service provision of biological soil crusts in the human-modified Caatinga. *Front Ecol Evol* 2019;7:482.

Tamm A, Caesar J, Kunz N *et al.* Ecophysiological properties of three biological soil crust types and their photoautotrophs from the Succulent Karoo, South Africa. *Plant Soil* 2018;429:127–146.

Vinoth M, Sivasankari S, Ahamed A *et al*. Biological soil crust (BSC) is an effective biofertilizer on *Vigna mungo* (L.). *Saudi J Biol Sci* 2020;27(9):2325–2332.

Wang L, Zhang G, Zhu L *et* al. Biocrust wetting induced change in soil surface roughness as influenced by biocrust type, coverage and wetting patterns. Geoderma 2017;306(15):1–9.

Williams W, Büdel B, Williams S. Wet season cyanobacterial N enrichment highly correlated with species richness and *Nostoc* in the northern Australian savannah, *Biogeosciences* 2018;15:2149–2159.

Williams W, Chilton A, Schneemilch M *et al*. Microbial biobanking – cyanobacteria-rich topsoil facilitates mine rehabilitation. *Biogeosciences* 2019;16:2189–2204.

Wu Y, Li X, Hasi-Eerdun *et al*. Surface roughness response of biocrust-covered soil to mimicked sheep trampling in the Mu Us sandy Land, northern China. *Geoderma* 2020;363:114146.

Yang X, Xu M, Zhao *Y et al*. Moss-dominated biological soil crusts improve stability of soil organic carbon on the Loess Plateau, China. *Plant Soil Environ* 2019;65:104-109.

Yeager CM, Kuske CR, Carney TD *et al*. Response of biological soil crust diazotrophs to season, altered summer precipitation, and year-round increased temperature in an arid grassland of the Colorado Plateau, USA. *Front Microbio* 2012. 3:358. doi:10.3389/fmicb.2012.00358.

Zaady E, Katra I, Yizhaq H. Inferring the impact of rainfall gradient on biocrusts’ developmental stage and thus on soil physical structures in sand dunes. *Aeolian Res* 2014;13:81-89.

Zhang X, Li J, Liu J *et al*. Temporal shifts in cyanobacterial diversity and their relationships to different types of biological soil crust in the southeastern Tengger Desert. *Rhizosphere* 2021;17:100322.

Zhang Y, Duan P, Zhang P *et al.* Variations in cyanobacterial and algal communities and soil characteristics under biocrust development under similar environmental conditions. *Plant Soil* 2017;429:241–251.

Zhao Y, Jia RL, Wang J. Towards stopping land degradation in drylands: Water-saving techniques for cultivating biocrusts in situ. *Land Degrad Dev* 2019;30:2336– 2346.

Zhao Y, Xu M, Belnap J. Potential nitrogen fixation activity of different aged biological soil crusts from rehabilitated grasslands of the hilly Loess Plateau, China. *J Arid Environ* 2010;74:1186-1191.

Zhou XB, Zhang YM, Yin BF. Divergence in physiological responses between cyanobacterial and lichen crusts to a gradient of simulated nitrogen deposition. *Plant Soil* 2016;399:121–134.

Zubiaga EB, Álvarez GH. Infiltración en biocostras en una región semiárida del centro de México. *Terra Latinoamericana* 2018;36:337-343.
