# Supplementary material for: Organizing a global list of cyanobacteria and algae from soil biocrusts evidenced great geographic and taxonomic gaps
Source: FEMS Microbiol Ecol. 2024 May 30;100(7):fiae086. doi: 10.1093/femsec/fiae086 (PMC11221558; doi:10.1093/femsec/fiae086)
Supplement: fiae086_Supplemental_Files [file fiae086_supplemental_files.zip › Supplementary Material S3.docx]

**Supplementary Material S3: Characteristics of the environments where each microalgae taxon was observed.**

| **Region** | **Substrate** | **Climate** | **Species** | **References** |
| --- | --- | --- | --- | --- |
| Temperate | Soil | Arid | *Bracteacoccus* sp.; *Chlorella* sp.; *Chlorella vulgaris*; *Chlorococcum* sp.; *Chloroidium*; *Desmococcus*; *Diplosphaera chodatii*; *Diplosphaera* sp.; *Klebsormidium* sp.; *Stichococcus* sp.; *Stichococcus*; *Trebouxia* sp. | Flechtner *et al.* 2009*; Zaady *et al*. 2014; Sorochkina *et al*. 2018; Condon *et al*. 2019*; Samolov *et al*. 2020; Zhao *et al*. 2021 |
|  |  | Semiarid | *Bracteacoccus* sp.; *Chlorella vulgaris*; *Chlorococcum* sp.; *Chloroidium;* *Desmococcus*; *Diplosphaera chodatii; Elliptochloris subsphaerica*; *Klebsormidium flaccidum*; *Stichococcus* sp.; *Trebouxia* sp. | Flechtner *et al*. 2009*; Zhao *et* al. 2010; Zaady *et al*. 2014; Samolov *et al*. 2020 |
|  |  | Mediterranean | *Bracteacoccus* sp.; *Chlamydomonas* sp.; *Chlorella* sp.; *Chloroidium*; *Diplosphaera chodatii*; *Desmococcus*; *Elliptochloris subsphaerica*; *Klebsormidium* cf. *nitens*; *Myrmecia bisecta*; *Trebouxia* sp. | Moya *et al*. 2020; Samolov *et al*. 2020 |
|  |  | Transitional oceanic to continental | *Chlamydomonas* sp.; *Chlorella vulgaris*; *Chlorococcum* sp.; *Chloroidium ellipsoideum*; *Diplosphaera chodatii*; *Myrmecia bisecta*; *Pseudochlorella*; *Stichococcus bacillaris*; *Tetracystis* sp. | Borchhardt MA *et al*. 2019; Gypser *et al*. 2016 |
|  |  | Humid | *Bracteacoccus minor*; *Bracteacoccus* sp; *Chlamydomonas* sp.; *Chlorella* sp.; *Chlorella vulgaris*; *Chlorococcum* sp.; *Chloroidium*; *Chloroidium ellipsoideum*; *Diplosphaera chodatii*; *Desmococcus*; *Elliptochloris subsphaerica*; *Klebsormidium* cf. *nitens*; *Klebsormidium flaccidum*; *Klebsormidium* sp.; *Myrmecia bisecta*; *Pseudochlorella*; *Stichococcus bacillaris*; *Tetracystis* sp.; *Trebouxia* sp. | Schulz *et al*. 2016; Samolov *et al*. 2020 |
|  |  | Low arctic | *Bracteacoccus* sp.; *Chlamydomonas* sp.; *Chlorella vulgaris*; *Chlorococcum* sp.; *Diplosphaera* sp.; *Heterococcus*/ *Elliptochloris subsphaerica*; *Klebsormidium flaccidum*; *Klebsormidium* cf. *nitens*; *Myrmecia*; *Myrmecia bisecta*; *Nannochloris* sp.; *Navicula*; *Nitzschia*; *Stichococcus*; *Tetracystis* sp. | Pushkareva *et al*. 2021; *Pombubpa* *et al*. 2020 |
|  |  | No information | *Achnanthidium minutissimum*; *Chlorella* sp.; *Chlorella vulgaris*; *Navicula* sp.; *Nitzschia palea*; *Trebouxia* sp. | Forster *et al*. 2006; Nowicka-Krawczyk *et al*. 2014 |
|  | Rock | No information | *Achnanthidium minutissimum*; *Acutodesmus obliquus* (Currently *Tetradesmus obliquus*); *Bracteacoccus* sp.; *Chlamydomonas*; *Chlorella* sp.; *Chlorella vulgaris*; *Nitzschia palea*; *Stichococcus bacillaris*; *Trebouxia* sp. | Hodac *et al*. 2015; Nowicka-Krawczyk *et al*. 2014 |
|  | No information | | *Achnanthidium; Achnanthidium minutissimum; Gomphonema*; *Naciula* sp.; *Nitzschia*; *Nitzschia palea*; *Pinnularia* sp. | Arp *et al*. 2010; Bailet *et al*. 2019; Bengtsson *et al*. 2018 |
| Tropical | Soil | Semiarid | *Chlorella* sp.; *Heterococcus*; *Stichococcus* sp.; *Klebsormidium* sp. | Szyja *et al*. 2019 |
| Polar | Soil |  | *Bracteacoccus*; *Chlamydomonas*; *Chlorococcum; Chloroidium*; *Desmococcus*; *Diplosphaera*; *Hantzschia*; *Heterococcus*; *Klebsormidium*; *Klebsormidium flaccidum;* *Pinnularia*; *Pseudochlorella*; *Myrmecia*; *Navicula*; *Nitzschia; Stichococcus*; *Stichococcus*; *Trebouxia* | Rippin *et al*. 2019; Rippin *et al*. 2018 |
| Extreme environments | | | *Bracteacoccus minor*; *Chlorella* sp.; *Chlorella vulgaris*; *Chlorococcum* sp.; *Chloroidium ellipsoideum*; *Diplosphaera chodatii*; *Elliptochloris subsphaerica*; *Myrmecia bisecta*; *Nannochloris* sp.; *Stichococcus bacillaris*; *Tetracystis* sp. | García-Meza *et al*. 2006; Sommer V *et al*. 2020^a^; Sommer V *et al*. 2020^b^ |
| No information | | | *Klebsormidium* sp.; *Chlamydomonas* sp. | Belnap, Büdel 2016; Caesar J *et al*. 2018; Zhang *et al*. 2017 |

* The article does not directly mention climate or habitat, but they can be inferred by other information present in the text.

Belnap J, Büdel B. Biological Soil Crusts as Soil Stabilizers. In: Weber B., Büdel B., Belnap J. (eds) Biological soil crusts: an organizing principle in drylands. Ecological studies (analysis and synthesis). Ed. Springer 2016;226.

Caesar J, Tamm A, Ruckteschler N *et al*. Revisiting chlorophyll extraction methods in biological soil crusts – methodology for determination of chlorophyll a and chlorophyll a + b as compared to previous methods. *Biogeosciences* 2018;15:1415–1424.

Condon LA, Pietrasiak N, Rosentreter R *et* al. Passive restoration of vegetation and biological soil crusts following 80 years of exclusion from grazing across the Great Basin. *Restor Ecol* 2019;28:S75-S85.

Flechtner VR, Johansen JR, Belnap J. The biological soil crusts of the San Nicolas Island: enigmatic algae from a geographically isolated ecosystem. *West N Am Nat* 2009;68(4):405-436.

Pushkareva E, Baumann K, Van AT *et al*. Diversity of microbial phototrophs and heterotrophs in Icelandic biocrusts and their role in phosphorus-rich Andosols. *Geoderma* 2021;386:114905.

Rippin M, Borchhardt N, Karsten U and Becker B. (2019) Cold acclimation improves the desiccation stress resilience of polar strains of *Klebsormidium* (Streptophyta). *Front. Microbiol.* 10:1730. doi: 10.3389/fmicb.2019.01730.

Samolov E, Baumann K, Büdel B *et al*. Biodiversity of Algae and Cyanobacteria in Biological Soil Crusts Collected Along a Climatic Gradient in Chile Using an Integrative Approach. *Microorganisms* 2020;*8*:1047.

Schulz K, Mikhailyuk T, Dreßler M *et al*. Biological soil crusts from coastal dunes at the Baltic Sea: cyanobacterial and algal biodiversity and related soil properties. *Microb Ecol* 2016;71:178–193.

Sommer V, Karsten U, Glaser K. Halophilic Algal Communities in Biological Soil Crusts Isolated From Potash Tailings Pile Areas. *Front Ecol Evol* 2020^a^;8:46.

Sommer V, Mikhailyuk T, Glaser K *et al*. Uncovering Unique Green Algae and Cyanobacteria Isolated from Biocrusts in Highly Saline Potash Tailing Pile Habitats, Using an Integrative Approach. *Microorganisms* 2020^b^;8:1667.

Sorochkina K, Ayuso SV, Garcia-Pichel F. Establishing rates of lateral expansion of cyanobacterial biological soil crusts for optimal restoration. *Plant Soil* 2018;*429*(1-2):199-211.

Szyja M, Menezes AGS, Oliveira FDA *et al*. Neglected but potent dry forest players: ecological role and ecosystem service provision of biological soil crusts in the human-modified Caatinga. *Front Ecol Evol* 2019;7:482.

Zaady E, Katra I, Yizhaq H. Inferring the impact of rainfall gradient on biocrusts’ developmental stage and thus on soil physical structures in sand dunes. *Aeolian Res* 2014;13:81-89.

Zhang Y, Duan P, Zhang P *et al.* Variations in cyanobacterial and algal communities and soil characteristics under biocrust development under similar environmental conditions. *Plant Soil* 2017;429:241–251.

Zhao K, Zhang B, Li J *et al*. The autotrophic community across developmental stages of biocrusts in the Gurbantunggut Desert. *Geoderma* 2021;388:114927.

Zhao Y, Xu M, Belnap J. Potential nitrogen fixation activity of different aged biological soil crusts from rehabilitated grasslands of the hilly Loess Plateau, China. *J Arid Environ* 2010;74:1186-1191.
